# Validation of Gazepoint low-cost eye-tracking and psychophysiology bundle

**DOI:** 10.3758/s13428-021-01654-x

**Published:** 2021-08-17

**Authors:** Hélio Clemente Cuve, Jelka Stojanov, Xavier Roberts-Gaal, Caroline Catmur, Geoffrey Bird

**Affiliations:** 1 Department of Experimental Psychology, University of Oxford, Oxford, UK; 2 Department of Psychology, Institute of Psychiatry, Psychology and Neuroscience, King’s College London, London, UK; 3 Social, Genetic and Developmental Psychiatry Centre, Institute of Psychiatry, Psychology and Neuroscience, King’s College London, London, UK

**Keywords:** Eye-tracking, Psychophysiology, Gaze position, Pupillometry, Skin conductance, Heart rate, Validation, Gazepoint GP3-HD

## Abstract

Eye-tracking and recording of physiological signals are increasingly used in research within cognitive science and human–computer interaction. For example, gaze position and measures of autonomic arousal, including pupil dilation, skin conductance (SC), and heart rate (HR), provide an indicator of cognitive and physiological processes. The growing popularity of these techniques is partially driven by the emergence of low-cost recording equipment and the proliferation of open-source software for data collection and analysis of such signals. However, the use of new technology requires investigation of its reliability and validation with respect to real-world usage and against established technologies. Accordingly, in two experiments (total *N* = 69), we assessed the Gazepoint GP3-HD eye-tracker and Gazepoint Biometrics (GPB) system from Gazepoint. We show that the accuracy, precision, and robustness of the eye-tracker are comparable to competing systems. While fixation and saccade events can be reliably extracted, the study of saccade kinematics is affected by the low sampling rate. The GP3-HD is also able to capture psychological effects on pupil dilation in addition to the well-defined pupillary light reflex. Finally, moderate-to-strong correlations between physiological recordings and derived metrics of SC and HR between the GPB and the well-established BIOPAC MP160 support its validity. However, low amplitude of the SC signal obtained from the GPB may reduce sensitivity when separating phasic and tonic components. Similarly, data loss in pulse monitoring may pose difficulties for certain HR variability analyses.

**Supplementary Information:**

The online version contains supplementary material available at 10.3758/s13428-021-01654-x.

## Introduction

Eye-tracking and psychophysiological recording^1^ have gained popularity in recent years as a way to gain insight into cognitive processes, particularly the time course of those processes (Cacioppo et al., 2016; Holmqvist et al., 2011). The use of these techniques for research is not new; attempts to track human gaze and link physiological signals (e.g., heart rate, skin conductance, and pupillary change) to cognition, although highly expensive and invasive, can be found even in the 19th century (see, Buswell, 1935; Cacioppo et al., 2016; Dodge & Cline, 1901 for a review).

While of interest to researchers for decades, this technology was until recently, mostly limited to research groups that could afford their high cost along with the proprietary software necessary to analyze the data (Funke et al., 2016). However, the proliferation of technology companies working on virtual reality, human–computer interaction, and marketing, has diversified research into, and applications of, eye-tracking and psychophysiology technologies.

Manufacturers are now starting to offer low-cost eye-trackers (e.g., GP3 and GP3-HD from Gazepoint; Tobii Eye Tracker 4C and Tobii Eye Tracker 5 from Tobii or the now discontinued EyeTribe), and there is increased availability of open-source data acquisition and analysis software (e.g., OpenSesame - Mathôt et al., 2012; PsychoPy - Peirce et al., 2019; PyGaze - Dalmaijer et al., 2014; GazeR - Geller et al., 2020). Similarly, there are several psychophysiology devices for skin conductance and heart rate measurement targeted at both consumers, e.g., Fitbit bracelets, Apple Watch, and smartphone apps (Mühlen et al., 2021), and researchers (e.g., Shimmer by Tobii, the E4 wristband by Empatica), alongside the more conventional (and more expensive) devices traditionally used for scientific research. A number of established open-source tools for analyses of signals like SCR (Ledalab, Benedek & Kaernbach, 2010; PSPM - Bach & Staib, 2015; EDAExplorer - Taylor et al., 2015) and heart rate and variability (ArtiFact, Kaufmann et al., 2011; Kubios - Tarvainen et al., 2014; RapidHRV; Kirk et al., 2021) have made it easier to automate often cumbersome pre-processing procedures.

These inexpensive eye-tracking solutions represent a very attractive option, not only for researchers operating on a limited budget, but also for those interested in more portable and less cumbersome eye-tracking devices that can be easily moved and retrofitted according to specific study purposes and environments. There are also several potential advantages provided by the newer and simpler devices to measure SC and HR. For instance, traditional psychophysiological recording takes time to set up and can be invasive (e.g., attaching specialized ECG sensors to the participant’s chest and torso, often requiring the removal of clothing), which can add burden particularly to special participant populations (e.g., clinical groups).

While the diversification of eye-tracking and psychophysiology solutions provides considerable opportunities, it can also represent a risk to research validity and reproducibility if researchers are not adequately informed about the limitations of the low-cost devices available on the market (Orquin & Holmqvist, 2018; Society for Psychophysiological Research Ad, 2012). For eye-tracking applications, manufacturers commonly specify spatial accuracy—the average distance between a known target in space and the gaze position estimated by the eye-tracker; and spatial precision—the average distance between consecutive gaze position data points where gaze is assumed to have remained relatively stationary (Holmqvist et al., 2012). However, manufacturers’ performance evaluations are usually conducted under optimal conditions with trained participants using chinrests, or even using artificial eyes (Hessels, Cornelissen, et al., 2015b). As a result, relying solely on performance estimates provided by the manufacturers can yield unjustified optimism when evaluating the suitability of low-cost eye-tracking devices to answer certain research questions. Aware of the need for performance evaluations in more realistic experimental conditions, eye-tracking researchers have conducted extensive validation and comparison studies for some of the most frequently used eye-tracking devices (see, Funke et al., 2016; Hessels, Andersson, et al., 2015a; Janthanasub & Meesad, 2015; Leube et al., 2017; Mannaru, Balasingam, Pattipati, Sibley, & Coyne, 2017b; Niehorster et al., 2018). A common observation across these studies is that, even under ideal conditions, there is still a great deal of variability in how well different eye-tracking systems perform.

In addition to gaze position, eye-movement researchers often study saccades. However, systematic analysis of saccades in validation studies is often overlooked. While system accuracy and precision can inform saccadometry research, there aren’t many established baselines for saccade metrics and most research has relied on direct comparison of different eye-trackers (e.g., Dalmaijer, 2014; Nyström, Niehorster, Andersson & Hooge, 2021). Nonetheless, it is possible to assess saccade parameters descriptively, for example, by looking at known regular relationships between saccade parameters (e.g., duration, amplitude, velocity) known as the saccadic ‘main sequence’ (Bahill et al., 1975; Gibaldi & Sabatini, 2021). The shape of the main sequence is well known—for small to medium saccades (between 10 and 20 degrees of visual angle in size), one should expect the relationship between these saccade metrics to be approximately linear (Gibaldi & Sabatini, 2021).

Similarly, for a given task where the size of the expected saccade is known, researchers could use the actual observed saccades of typical participants to assess undershooting or overshooting, as well the degree of saccade curvature (van Leeuwen & Belopolsky, 2018).

In this study, we aimed to assess the performance of a new relatively low-cost eye-tracker, the GP3-HD (Gazepoint), with a sampling rate of 150 Hz and incorporating a high-definition machine vision-powered camera. The GP3-HD replaces the previous model, the GP3, which recorded at 60 Hz and for which independent validations exist (Brand et al., 2020; Mannaru, Balasingam, Pattipati, & Sibley, 2017a).

In addition to the GP3-HD, Gazepoint recently launched a Biometrics system (GPB) for the measurement of autonomic responses, specifically, skin conductance (SC) and heart rate (HR). SC and HR provide an indication of the degree of an individual’s physiological arousal, and the physio-anatomical mechanisms underlying changes in SC and HR are relatively well understood. As is the case with the GP3-HD, however, the reliability and validity of the GPB is currently unknown. A comparison of raw and derived SC and HR metrics obtained from the GPB and from a well-established device would provide a useful insight into the potential of the GPB to provide valid measurements. Therefore, this study also aimed to validate the GPB.

In Experiment 1, common data quality indicators (calibration quality, data loss, accuracy, precision) were obtained for the GP3-HD eye-tracker. We also provide information on sampling rate variability and fixation and saccade metrics (Holmqvist et al., 2011). In addition to gaze position and saccade analyses, we provide pupillometry analyses tracking the pupillary light reflex (PLR), a physiological process in which the pupil constricts in response to increased light intensity and dilates in response to reduced light intensity (Mathôt, 2018). In Experiment 2, we provide a second validation of the GP3-HD system that enabled us to study its performance under the conditions encountered in a typical psychological experiment, as well as to further test measurement of pupillary responses. Additionally, in Experiment 2, data were collected simultaneously from a well-established psychophysiological recording system (BIOPAC-MP160) and from the GPB system to assess the validity of SC and HR data, and derived metrics, recorded from the GPB system. Finally, recommendations for researchers planning to use this technology are provided.

## Experiment 1

### Method

#### Participants

A total of 13 university students (seven women, 13 right-handed) took part in Experiment 1 after exclusion of one participant due to a failure to calibrate, and one for excessive data loss and difficulties tracking. They ranged in age from 19 to 28 years (M = 22.08, SD = 2.40). All participants had normal or corrected-to-normal vision and reported being able to complete the study without relying on vision correction. Hence, no participants wore glasses or contact lenses throughout the experiment. Finally, no participants wore make-up during the experiment.

#### Apparatus and task environment

The experiment was run on a Dell computer (Intel Core i7-3610QM @ 2.30 GHz, 16 GB RAM, Windows 10) and the task stimuli were presented on a monitor (53 x 30 cm, 60 Hz refresh rate, 1920 x 1080 pixels, 45.99 x 27.01 degrees of visual angle). The experiment was completed in a dimly lit, sound-proof testing room.

#### Eye-tracking

The remote GP3-HD eye-tracker, recording at 150 Hz, was used in this study. The eye-tracker was controlled through a custom script in PsychoPy (Peirce et al., 2019). The eye-tracker was placed at a 45° angle and 60–65 cm from the participants’ eyes (M_DISTANCE_ = 62.45 cm), in line with the instructions provided in the Gazepoint manual. The Gazepoint control and monitoring window, and physical measurement (before and between tasks) were used to aid setup and find an optimal position. Eye-tracker specifications provided by the manufacturer are summarized in Table 1.

**Table 1:** GP3-HD eye-tracker specifications offered by the manufacturer (Gazepoint)

GP3-HD specifications	Values
Accuracy	0.5–1^o^
Headbox	35 x 22 cm
Precision	NS
Price	$1995 (Hardware only) $995 (Professional software) $1495 (UX software)
Sampling rate	60 or 150 Hz
Tracking distance	50–100 cm (65 cm recommended)

Notes*. NS *Not specified by the manufacturer

Prior to starting the main tasks, calibration was performed using a nine-point grid followed by a validation sequence. Satisfactory calibration criteria for continuing with the task were determined *a priori*: (a) all nine calibration points had to be deemed valid according to the Gazepoint Control software; (b) average calibration error had to be below or equal to 40 pixels (approximately 1 degree of visual angle); and finally, (c) using a real-time gaze relay nine-point grid, where participants’ gaze was shown as moving green dots on the screen, participants were asked to report how good they thought the eye-tracker was at approximating where they were actually looking using a 0–10 scale after explicitly attending to each of the gaze targets. Only answers equal to, or above, 8 were accepted.

#### Tasks

##### Fixation-Saccade task

**Fig. 1:**
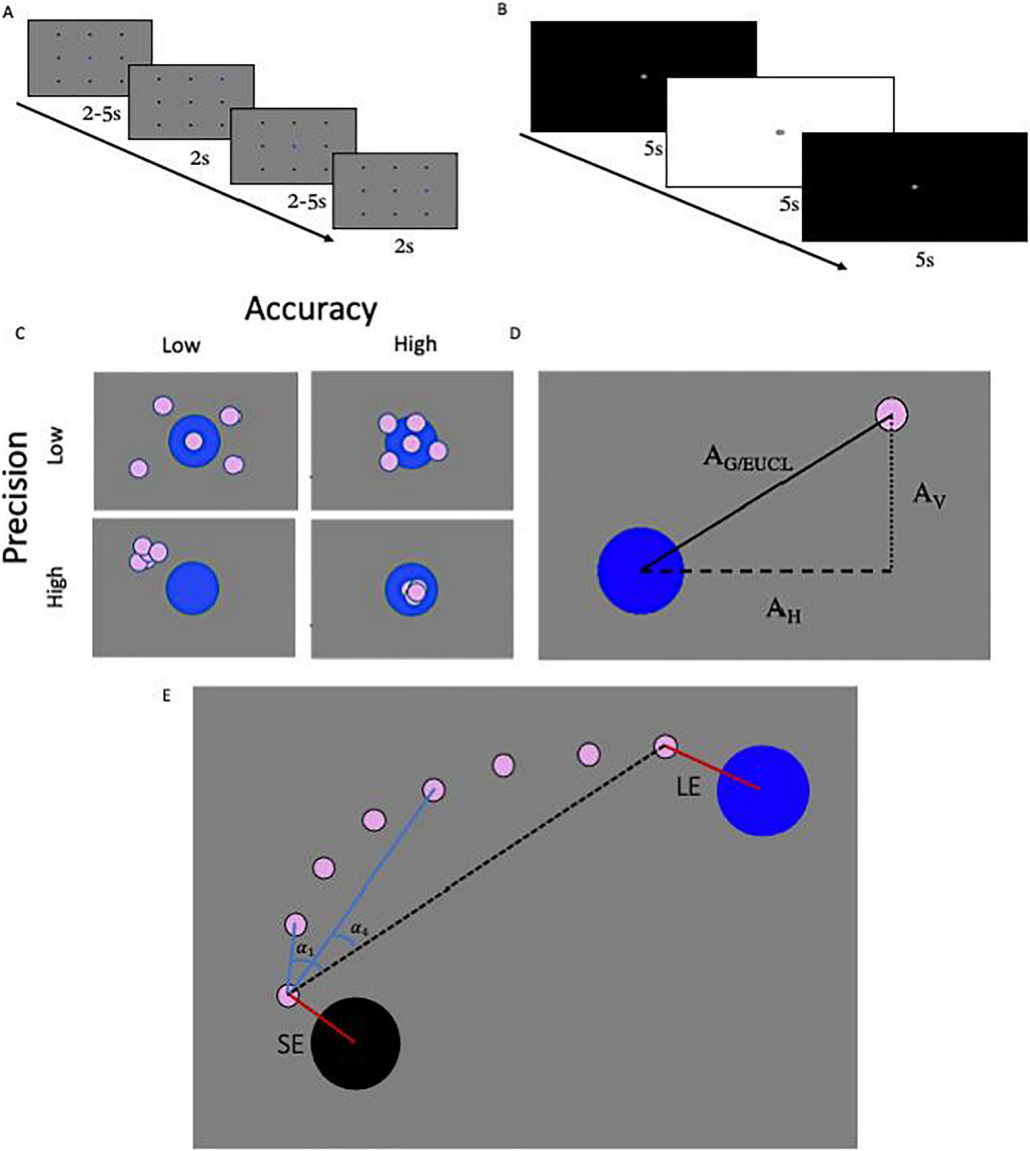
**a** Schematic of the Fixation-Saccade task. A blue target appeared and switched from the central dot to each of the peripheral dots. **b** Schematic of the Pupillary Light Reflex task. Each screen appeared 12 times. Both tasks were repeated twice, once with, and once without, a chinrest. **c** Illustration of the concepts of accuracy and precision for eye-tracking data. The *blue center dot* represents the known gaze target, and the *pink smaller circles* represent gaze locations estimated by the eye-tracker. **d** Three types of eye-tracker accuracy calculations. A_H_ = Horizontal accuracy for a particular gaze sample. A_V_ = Vertical accuracy for a particular gaze sample. A_G/EUCL_ = Global/Euclidian accuracy for a particular gaze sample. **e** Illustration of saccade metrics. SE = Starting error. LE = Landing error. Curvature = Median of all individual α_i_ angles between each sample point and the straight line connecting the start and end point of the saccade

Participants completed two main tasks. The Fixation-Saccade task was designed to provide data for the calculation of fixation and saccade metrics, and for accuracy and precision analyses. Participants were presented with nine black dots on the screen (size: 40 pixels, approximately 1 degree of visual angle; with an average distance of 11 degrees between target dots; see Fig. 1a). A target (blue dot) started on the central dot and then transitioned from the center to each peripheral dot at random throughout the task. All target positions were sampled before any were repeated. The duration of time for which the central dot was blue was varied between 2 and 5 s on each transition to prevent participants from trying to predict when and where the dot will move next. The task finished when each peripheral dot had turned blue twice.

##### Pupillary light reflex task

The second task was designed to evoke the pupillary light reflex (PLR). Participants were presented with a grey dot in the middle of the screen (size: 50 pixels, approximately 1.2 degrees of visual angle) and instructed to fixate on it while black and white backgrounds^2^ interchanged every 5 s. Each screen was presented 12 times (24 changes of color, see Fig. 1b).

### Procedure

Participants completed the setup and the calibration procedure, followed by the tasks detailed above. Each task was completed twice, once with the participants’ heads placed on the chinrest to limit their head movements, and once without. In both conditions participants were asked to avoid head and body movements. The order of tasks and conditions (chinrest vs. no-chinrest) was counterbalanced. The calibration procedure was performed prior to each task. After the experiment participants were debriefed. All experimental procedures were conducted in accordance with the revised 2013 Declaration of Helsinki and were approved by the local research ethics committee.

#### Pre-processing

Eye-tracking data were pre-processed using custom code in R (version: 3.6.1). Gaze samples falling outside screen coordinates were eliminated, as well as the samples labeled as invalid by the eye-tracker (in total, 2.72% of the samples were excluded, out of which 0.74% fell outside the screen boundaries, and 1.98% were labeled as invalid by the eye-tracker). A simple implementation of the adaptive velocity-based algorithm proposed by Engbert and Kliegl (2003) was used to detect fixations and saccades in the ‘Fixation-Saccade’ task. Saccades were defined as periods of at least 20 ms (the duration of three adjacent gaze samples) where velocity exceeded an adaptive threshold set for each participant and condition (chinrest and no-chinrest) based on the level of noise in the data. The velocity threshold was defined as 5 median absolute deviations above the median velocity for each participant and condition. Finally, to prevent artificial improvements in accuracy and precision, no smoothing, filtering, or interpolation was applied to gaze position coordinates.

For the PLR task, pupil data were first cleaned by removing pupil sizes outside the range of 2–10 mm. Pupil data were then pre-processed using functions from the R package GazeR (Geller et al., 2020). Data loss (e.g., blinks) up to 150 ms in duration were imputed using linear interpolation. Finally, a subtractive baseline correction was applied in line with the recommendations in Mathôt et al. (2018). Median pupil size during the last 20 samples of the preceding trial and the first 20 samples of the current trial (approximately 240 ms, incorporating an equal duration of light and dark screens) was taken as baseline pupil size, from which all individual pupil sizes were subtracted on each trial.

#### Metrics and analyses

##### Calibration quality

To assess the calibration quality, two metrics were used based on the manufacturer’s calibration procedure: 1) the number of calibration attempts it took until the experimenter accepted the calibration, and 2) the average error of the accepted calibration. All calibrations needed to have nine valid calibration points to be accepted so the number of valid calibration points was not considered in further analyses.

##### Sampling rate variability

As the GP3-HD eye-tracker has a sampling frequency of 150 Hz, the expected average inter-sample time is approximately 0.0067 s (6.7 ms). Sampling rate variability was assessed by calculating the mean and the standard deviation of inter-sample time as well as their robust equivalents (median and median absolute deviation), for both the chinrest and no-chinrest condition. Sampling rate variability was assessed across both the Fixation-Saccade and the PLR task.

##### Data loss

Data loss occurs when the eye-tracker cannot detect the position of the eyes, and individual samples where this happens are labeled as invalid by the device. Proportion of lost gaze was computed for each trial and participant and compared between conditions (chinrest and no-chinrest).

##### Accuracy

Prior to computing accuracy and precision, the first and last 250 ms of each trial were removed to give participants time to fixate on the new target dot and to limit the extent to which participants’ anticipatory saccades influenced these metrics. This interval was chosen after calculation and visual inspection of saccade latency. Accuracy was computed as the error between the estimated gaze location and the location of a known target (Holmqvist et al., 2012). Horizontal and vertical accuracy were calculated for each gaze sample in the Fixation-Saccade task by subtracting estimated *x* and *y* gaze coordinates from the pre-defined *x* and *y* coordinates of each target dot location. Sample-level global accuracies were calculated as Euclidian distances between estimated *x* and *y* gaze coordinates and pre-defined x and y coordinates of target dot locations (see Fig. 1c, d).

Having calculated all three types of sample-level accuracies, outlier samples were removed if they were greater than 4 median absolute deviations from the median respective accuracy for each participant, condition, and trial (2.1% of the samples were excluded for vertical accuracy, 3% for horizontal accuracy, and 2.9% for global accuracy—note that these values are not independent). These outliers corresponded mostly to saccades, with the size of the error matching the expected saccade sizes during the task (note that analyses with outliers yielded consistent results, see Tables S1 and S2 in Supplementary materials). This was performed to avoid biasing the accuracy calculation by including gaze samples where the participant was likely to have clearly moved their eyes away from the target dot. Finally, mean vertical, horizontal, and global accuracy were calculated for each participant, condition, and trial.

Following the calculation of descriptive statistics, linear mixed models in lme4 (Bates et al., 2014) were fitted to test whether global accuracy differed between conditions (chinrest and no-chinrest) and target dot locations (central and peripheral) while accounting for participant and trial random effects. Maximal models were always fitted first (Barr et al., 2013), and convergence and singularity warnings were resolved by simplifying the random structure using principal component analysis to determine the most relevant random components (Bates et al., 2015).

Finally, we decided to compare accuracy on the central dot location against accuracy on all the peripheral dot locations grouped together for two reasons: (a) in psychological research, stimuli are commonly presented at the center of the screen, and it might be useful for researchers to know whether accuracy at this location is superior to accuracy at any peripheral location; (b) since trials with different target dot locations varied in frequency and duration (central target was presented more frequently and for a longer period of time in comparison to peripheral targets), grouping all peripheral locations allowed us to increase statistical power. Additionally, only sample-level accuracies within the first 2 s of the central trials were used in this comparison in order to match their duration with the duration of peripheral trials. For analyses, accuracy was log-transformed to correct the violation of the assumption that the residuals of the model are normally distributed.

##### Precision

Precision is a measure of the spatial variance in accuracy when the eye is assumed to be relatively stationary (Holmqvist et al., 2012). Therefore, gaze samples were first parsed into fixations and saccades based on the adaptive velocity threshold described above. Only fixations longer than 80 ms were used for calculating precision to avoid including small saccades which may be inaccurately labeled as fixations. Horizontal and vertical precision were calculated on a trial-by-trial basis for each participant by computing the root mean square from successive gaze samples for each fixation to the target dot. Global precision was calculated by first estimating the Euclidian distances between pairs of adjacent gaze samples and then computing the root mean square over these distances. After calculating descriptive statistics, linear mixed models were fitted to test whether the three types of precision (horizontal, vertical, global) varied between conditions (chinrest and no-chinrest) and target locations (central and peripheral) while controlling for participant and trial variability. Comparison between central and peripheral target locations as well as the choice of model’s random structure followed the same logic as the analysis of accuracy. Precision data was also log-transformed for analysis due to non-normality of residuals.

##### PLR

Following pupil pre-processing and baseline correction, the degree of PLR elicited by the changing stimulus was estimated (i.e., pupil constriction in response to light and pupil dilation in response to darkness). More specifically, linear mixed models were fitted for each condition (chinrest and no-chinrest) to compare pupil size changes in response to the two stimuli (black vs. white).

##### Saccade metrics

Saccade starting and landing error, amplitude, gain, curvature, latency, mean, and peak velocity were calculated (see Fig. 1). These metrics are provided to allow researchers to judge how well saccade parameters are reflected in gaze data from the GP3-HD, as there are no standard norms to which these values can be judged against, other than direct comparisons with other systems.

Saccade starting error was calculated as the Euclidian distance between the gaze sample labeled as the saccade onset and the center of the starting target, while saccade landing error was calculated as the Euclidian distance between the gaze sample labeled as the saccade end and the target center (Dalmaijer, 2014). After calculating saccade amplitude (size of the saccade in degrees of visual angle), we proceeded to compute gain, the ratio between the observed saccade amplitude and the expected saccade amplitude, actual distance between the two consecutive target locations (Noto & Robinson, 2001). Saccades with gains less than 1 were too small (*hypometric*), while saccades with gains higher than 1 were too large (*hypermetric*). Gain provides an approximation of over- or under-estimation of the expected saccade amplitudes.

In order to capture saccade trajectories, curvature was calculated as the median angle between each gaze point in a saccade and an imaginary straight line connecting the start and the end of the saccade, following the strategy of van Leeuwen and Belopolsky (2018). Saccade latency is the time from target onset until the initiation of the saccade to that target.

Finally, velocity was calculated for each gaze sample making up a saccade by dividing inter-sample distance (in degrees of visual angle) by inter-sample time (in seconds), after which mean and peak velocity were computed for each saccade as a whole.

### Results

#### Calibration quality

**Fig. 2:**
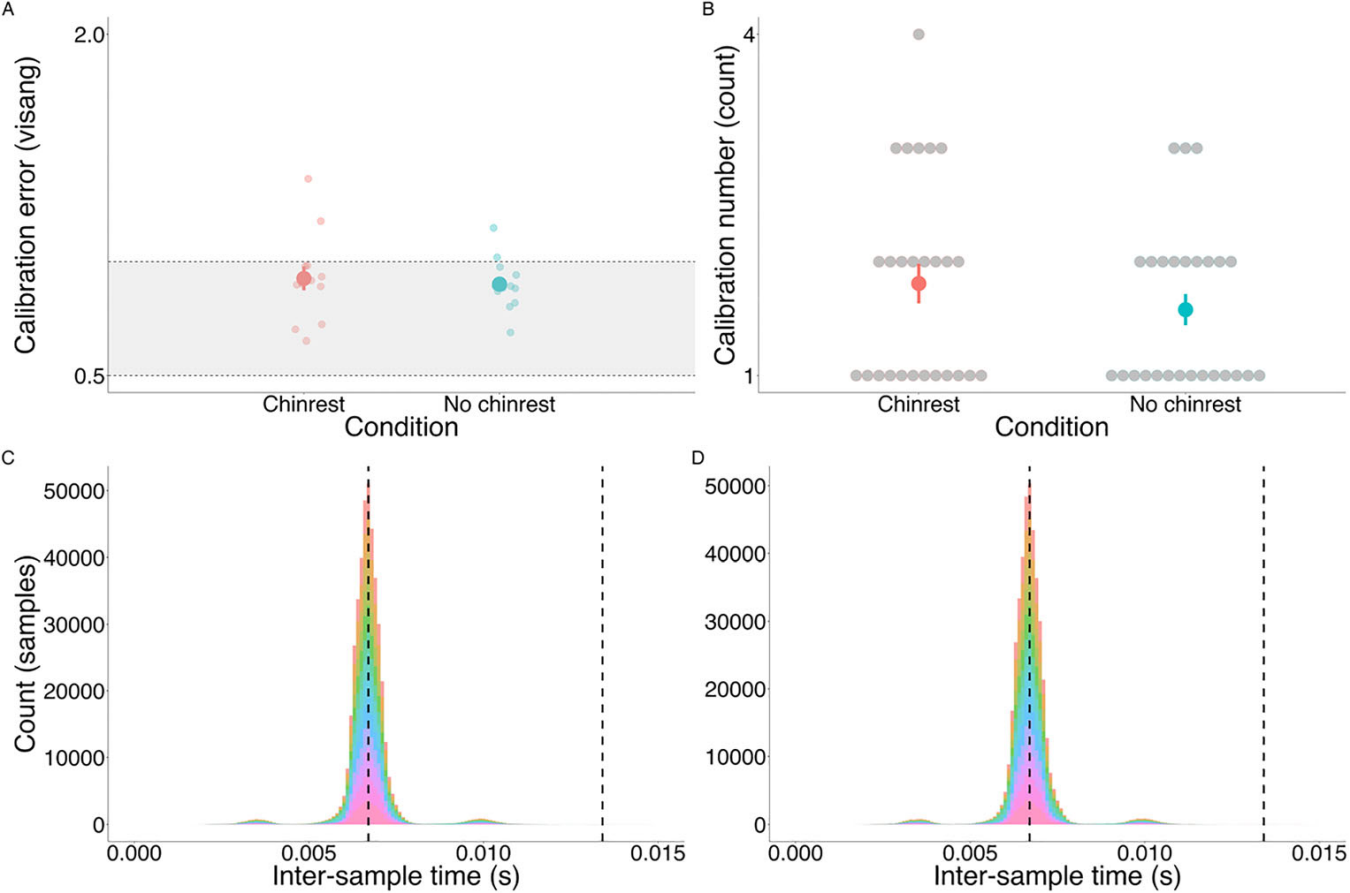
**a** Average error of the accepted calibration in degrees of visual angle. The *shaded part* indicates average accuracy stated by the manufacturer (0.5–1 degrees of visual angle). **b** The number of calibration attempts before the calibration was accepted. Each participant went through the calibration twice in each condition. **c** Distribution of the inter-sample times grouped in 0.0001 s (0.1 ms) bins in the chinrest condition, **d** and the no-chinrest condition. *Different colors* represent different participants (*N* = 13). The *dashed line on the left* indicates the expected inter-sample time based on the eye-tracker’s sampling rate (150 Hz), and the *dashed line on the right* indicates the duration of two consecutive samples

Calibration metrics included the number of attempts it took to achieve an acceptable calibration, and the average error of the accepted calibration. Due to the violation of normality (Shapiro–Wilk test: *W*(12) = 0.72, *p* < .001), a Wilcoxon signed-rank test was performed to examine differences between the chinrest and no-chinrest conditions on both calibration quality metrics. No differences were detected in the number of calibration attempts (M_CHINREST_ = 1.81, M_NOCHINREST_ = 1.58, *W*(12) = 30, *p* = .402) nor in the average error of the accepted calibration (M_CHINREST_ = 0.93^3^, M_NOCHINREST_ = 0.99, *W*(12) = 47, *p* = .946) (see Fig. 2).

#### Sampling rate variability

As expected, the observed average inter-sample time was 6.7 ms and the standard deviation of the inter-sample time was 0.79 ms (robust descriptives: Mdn = 6.68 ms; MAD = 35.29 ms). Only 0.0003% of the inter-sample times were greater than the duration of two consecutive samples (13.4 ms). Distribution of inter-sample time is shown in Fig. 2.

#### Data loss

A Wilcoxon signed-rank test was performed to compare whether the chinrest and no-chinrest condition differed in the proportion of lost gaze across tasks, and no differences were observed (M_CHINREST_ = .031, M_NOCHINREST_ = .029, *W*(12) = 43, *p* = .583) (see Fig. S4 in the Supplementary materials). Additionally, the quality of the accepted calibration prior to each combination of condition and task (Fixation-Saccade task – chinrest condition; Fixation-Saccade task – no chinrest condition; pupil task – chinrest condition; pupil task – no chinrest condition) did not correlate with the proportion of lost gaze (Kendall’s tau: t_FIX_SACC_CHINREST_ = –.24, *p* = .228; t_FIX_SACC_NO_CHINREST_ = .01, *p* = 1; t_PUPIL _CHINREST_ = .01, *p* = 1; t_PUPIL_NO_CHINREST_ = –.21, *p* = .331).

#### Accuracy

**Fig. 3:**
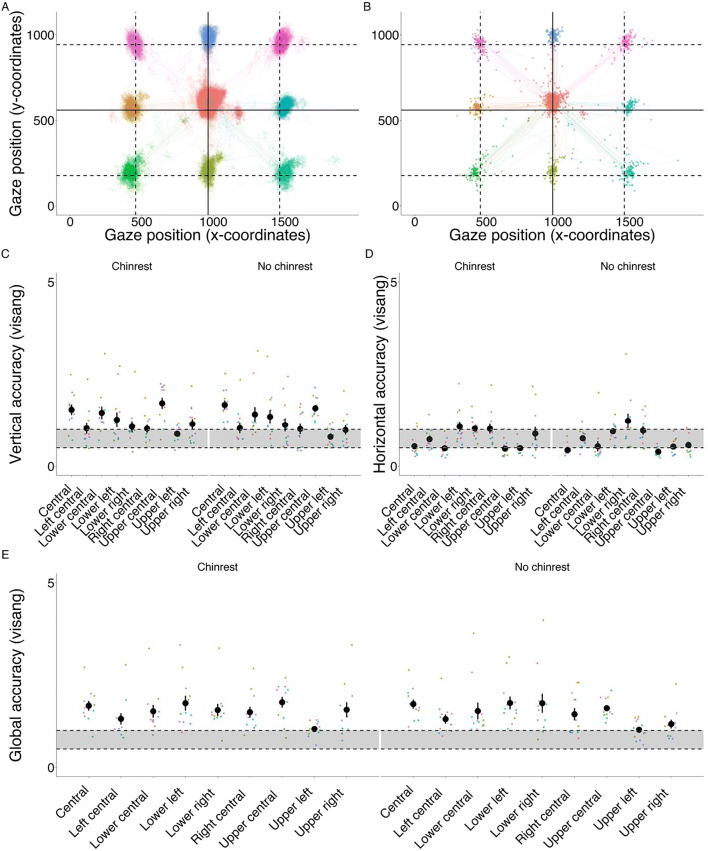
**a** Estimated gaze locations at each target location (individual gaze samples plotted). **b** Estimated gaze locations at each target dot location (mean fixation positions plotted). **c** Vertical, **d** horizontal, and **e** global accuracy in degrees of visual angle for each condition (chinrest, no-chinrest) and target dot location in the Fixation-Saccade task. The *shaded part* indicates average accuracy indicated by the manufacturer (0.5–1 degrees of visual angle). *Colored dots* represent participants’ accuracy in each iteration of target location, while the *black dot* and *error bars* represent means and 95% CIs

A visualization of participants’ gaze positions superimposed on target positions for the Fixation-Saccade task is provided in Fig. 3. No differences in global accuracy, defined as the Euclidian distance of the actual gaze sample from the expected gaze position, were found between conditions with and without the chinrest (estimate < 0.01, SE = 0.01, *t* = – 0.37, *p* = .710), while peripheral target locations had slightly better global accuracy in comparison to the central position (estimate = – 0.09, SE = 0.02, *t* = – 4.29, *p* < .01). However, different accuracy profiles were observed when vertical and horizontal accuracies were analyzed separately. In the case of vertical accuracy, no differences were found between conditions (estimate = 0.02, SE = 0.01, *t* = 1.64, *p* = .102), while vertical error at the peripheral target locations was lower than at the central target location (estimate = – 0.19, SE = 0.03, *t* = – 6.79, *p* = < .001). In the case of horizontal accuracy, better accuracy was achieved without the chinrest (estimate = – 0.04, SE = 0.01, *t* = – 3.16, *p* < .01), and peripheral target locations had worse horizontal accuracy in comparison to the central target location (estimate = 0.16, SE = 0.03, *t* = 4.95, *p* < .001). Descriptive statistics for vertical, horizontal, and global accuracy for each condition and target location can be seen in Fig. 3 and Table 2.

**Table 2:** Descriptive statistics for accuracy and precision

	Accuracy			Precision		
Target dot	Vertical	Horizontal	Global	Vertical	Horizontal	Global
Chinrest						
Central	1.60	0.60	1.74	0.28	0.24	0.36
Left	0.99	0.77	1.31	0.27	0.25	0.36
Lower central	1.63	0.68	1.75	0.33	0.28	0.43
Lower left	1.27	1.21	1.84	0.25	0.28	0.37
Lower right	1.20	1.29	1.89	0.31	0.31	0.43
Right	1.08	1.27	1.76	0.3	0.25	0.38
Upper central	1.67	0.49	1.72	0.28	0.23	0.35
Upper left	0.88	0.49	1.04	0.28	0.24	0.36
Upper right	1.15	0.86	1.54	0.27	0.22	0.34
No chinrest						
Central	1.71	0.46	1.76	0.28	0.25	0.36
Left	1.00	0.81	1.33	0.26	0.26	0.36
Lower central	1.45	0.55	1.56	0.25	0.25	0.35
Lower left	1.29	1.00	1.74	0.25	0.29	0.38
Lower right	1.19	1.21	1.75	0.24	0.27	0.36
Right	1.06	0.96	1.47	0.24	0.25	0.35
Upper central	1.65	0.41	1.68	0.28	0.23	0.35
Upper left	0.87	0.56	1.08	0.27	0.23	0.35
Upper right	1.04	0.58	1.22	0.28	0.24	0.36

*Notes*. Vertical, horizontal and global accuracy and precision in degrees of visual angle averaged across participants for each target dot location and condition

These results suggest that globally, the accuracy of the GP3-HD is closer to the upper bound of the expected ~ 0.5–1° values, although horizontal accuracy is consistently closer to 0.5°. Nonetheless, the range of accuracy values is similar to what has been reported in previous evaluations of commercial eye-trackers (Funke et al., 2016; Holmqvist, 2017). Overall, the GP3-HD shows precision below 0.5°, which is also in line with what is reported using similarly priced eye-trackers and even some high-end systems (Funke et al., 2016; Holmqvist, 2017).

### Precision

No differences were detected between central and peripheral target locations in any of the precision metrics (Horizontal: estimate = 0.01, SE < 0.01, *t* = 1.86, *p* = .069; Vertical: estimate < 0.01, SE < 0.01, *t* = – 1.07, p = .288; Global: estimate < 0.01, SE = 0.01, *t* = 0.52, *p* = .607), whereas the differences between chinrest and no-chinrest conditions showed a less consistent pattern. The no-chinrest condition yielded increased vertical precision (estimate = – 0.01, SE < 0.01, *t* = – 2.66, *p* < .01), whereas no differences were observed in horizontal (estimate < 0.01, SE < 0.01, *t* = 1.53, *p* = .126) and global precision (estimate < 0.01, SE < 0.01, *t* = – 0.80, *p* = .426). Descriptive statistics for vertical, horizontal, and global precision for each condition and target location can be seen in Table 2.

Bayesian equivalents for condition comparisons are provided in the Supplementary materials – see Bayesian analyses for condition comparisons.

#### Pupillary light reflex

**Fig. 4:**
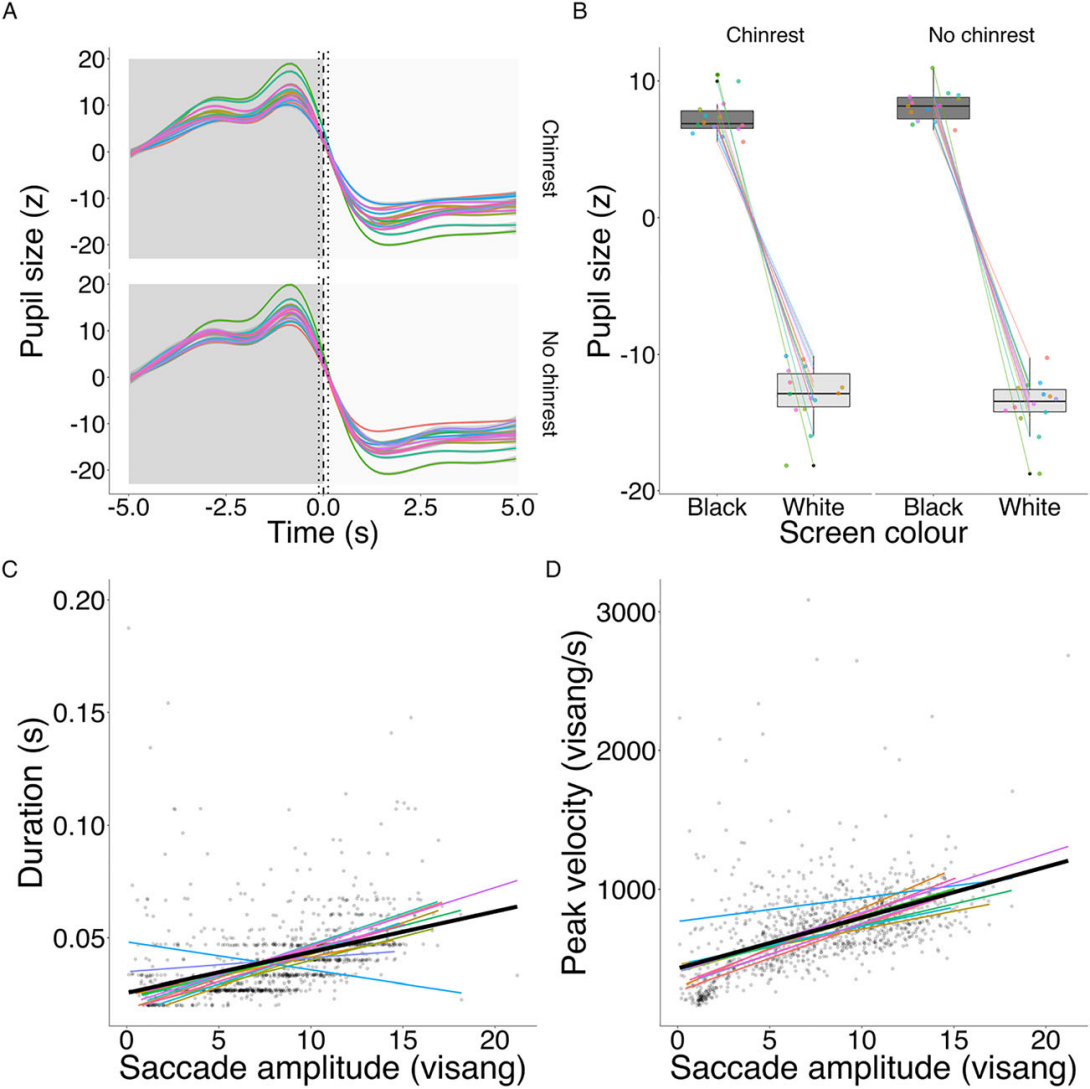
**a**, **b** Pupillary light reflex in both chinrest and no-chinrest conditions. *Dark-colored time section* (from – 5 to 0 s) and *dark boxplot* indicate pupil dilation during the black screen, *while light-colored time section* (from 0 to 5 s) and *light boxplot* indicate pupil constriction during the white screen. *Dashed line* in graph A indicates the switching point from black to white screen, while the *dotted lines* mark the baseline period used for calculating baseline pupil size. **c**, **d** Main sequence of saccadic eye movements (*r* = .288, *p* < .001; *r* = .420, *p* < .001). Each *colored line* represents a slope for each individual participant, while the *black line* represents the slope across participants. *Jittered points* presented in the background represent individual saccades

The PLR was reliably detected in both the chinrest (estimate = – 19.55, SE = 0.26, *t* = – 74.50, *p* < .001) and no-chinrest conditions (estimate = – 20.75, SE = 0.30, *t* = – 69.79, *p* < .001), see Fig. 4a, b). As expected, the PLR effect is very large, accounting for 95% of the variance in both conditions.

#### Saccadometry

Descriptive statistics for different saccade metrics are provided in Table 3. The relationships between saccade amplitude, duration and peak velocity are visualized in Fig. 4c, d.

**Table 3:** Saccade metrics calculated using GP3-HD data

Saccade index	GP3-HD M(SD)	Unit
Amplitude	8.87 (3.97)	Degrees of visual angle
Curvature	30.74 (12.94)	Degrees
Duration	48.80 (25.03)	Milliseconds
Gain	0.73 (0.21)	-
Latency	163.38 (369.75)	Milliseconds
Mean velocity	315.83 (95.73)	Degrees per second
Peak velocity	756 (322.48)	-
Starting error	3.56 (1.96)	Degrees of visual angle
Landing error	2.16 (2.28)	-

As expected, since the majority of the saccades detected in the Fixation-Saccade task would be classified as small using the guidelines of Gibaldi and Sabatini (2021), the relationship between these metrics was approximately linear.

**Fig. 5:**
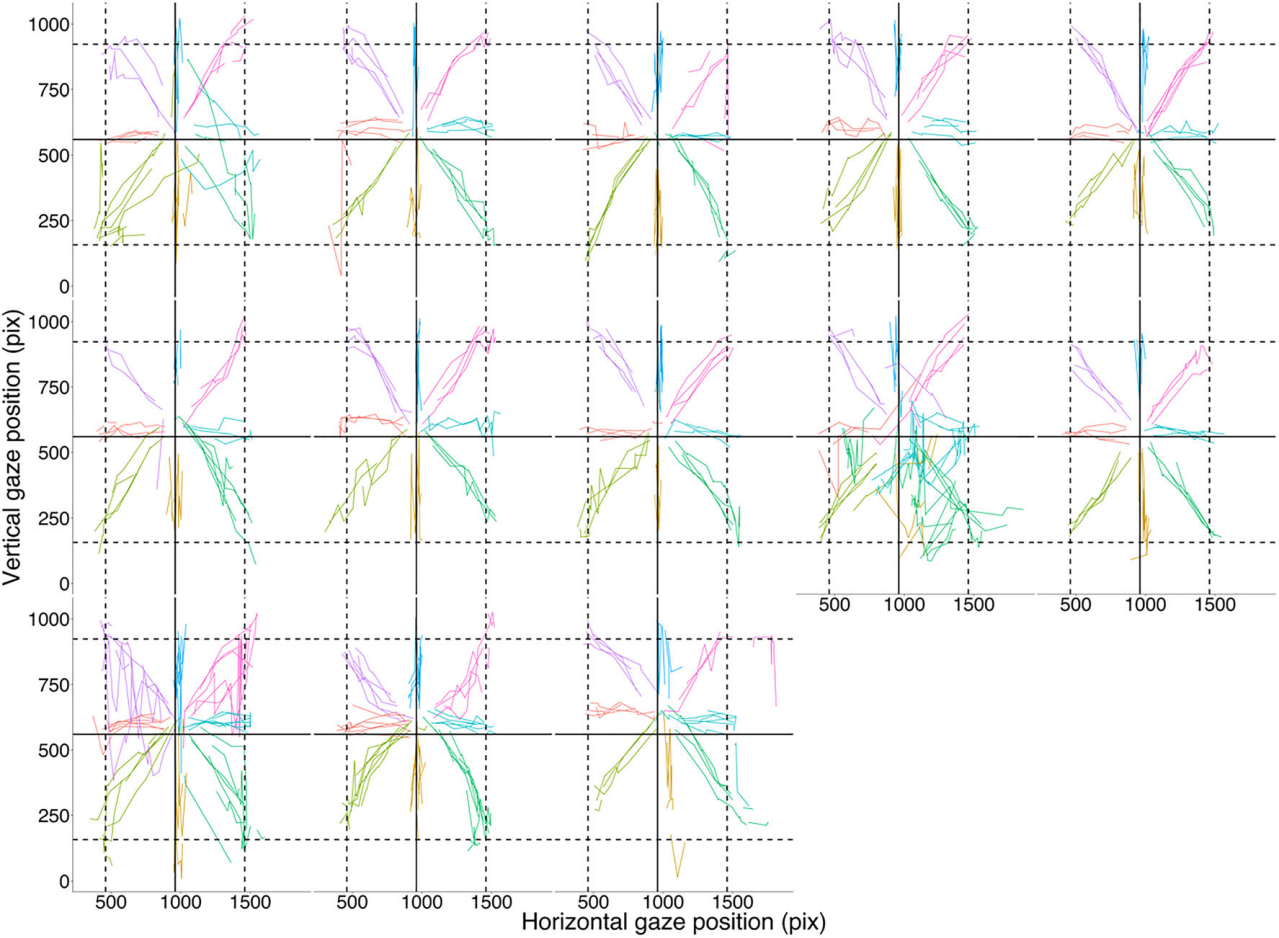
Saccade trajectories from the central target to each peripheral target. Each graph shows saccade trajectories of a different participant (*N* = 13; pix = pixel)

Finally, we examined each participant’s saccade trajectories from the central target to each peripheral target (see Fig. 5). Individual saccade trajectories plotted in Fig. 5 are not smooth, but appear broken and edgy, which is a sign of under-sampling (Dalmaijer, 2014). While clear identification of saccade events is possible, this suggests that the GP3-HD eye-tracker is less suitable for saccadometry research.

In summary, accuracy measures are comparable to the range reported in the existing eye-tracking literature for similar grade devices. In both ideal (chinrest) and non-ideal (no-chinrest) conditions, overall accuracy was at the upper limit or even higher than the values stated by the manufacturer (1 degree of visual angle). While this degree of accuracy is perfectly capable of capturing gaze behavior reliably across most experimental tasks, tracking targets smaller than this error could be problematic. While clear separation of saccades and fixations is possible using the GP3-HD, study of the properties of saccade kinematics is compromised by the low sampling rate.

### Discussion

Experiment 1 provided a standard validation of the GP3-HD eye-tracker. Overall, calibration and data loss were acceptable, with calibration achieved successfully for most participants after one or only a few attempts, and with a low rate of data loss over the experiment, comparable to more expensive eye-trackers based on previous reports (Holmqvist et al., 2011). Accuracy and precision of gaze tracking were also acceptable, but closer to upper desired limits (~1 degree of visual angle). This means that during stimulus design and presentation, researchers should accommodate this tracking error by ensuring that target locations are separated by 3 degrees of visual angle or more, so that if a participant was looking exactly at the middle of two targets, the estimated gaze locations (even accounting for error) would not fall onto either of the targets. It is worth noting, however, that better accuracy values were achieved (0.5 degrees of visual angle) particularly for the horizontal dimension. This is useful to know, as it is possible to calibrate how gaze is assigned to areas of interest given the tracking error associated with a particular participant at a particular point in time (Hessels & Hooge, 2019; Orquin & Holmqvist, 2018). Similarly, individualized accuracy and precision profiles can be used to calibrate gaze parsing filters (see, Feit et al., 2017).

With regard to saccade metrics, while the identification of saccades appears good, the calculation of kinematic parameters appears to be affected by the low sampling rate. As a result, the study of the properties of saccades using the GP3-HD may lead to inaccuracies depending on the specific parameters being studied. For example, it appears that the amplitude of saccades is underestimated, as the gain value was < 1, indicating hypometric saccades, that is, saccades where the amplitude was smaller than expected. Looking at the plots in Fig. 5, it is apparent that saccade patterns show breaks as a result of the sampling rate. This is consistent with Nyquist's theorem, which specifies that a signal must be sampled at more than twice the highest frequency component of the signal. Note, however, that saccade detection as well as peak velocity approximations are reliable with even 60 Hz sampling, and there are saccade reconstruction techniques that can improve the approximation of the “true” parameters of saccades (Wierts et al., 2008).

The lack of consistent significant differences between chinrest and no-chinrest conditions suggests that the algorithm for gaze estimation used by GP3-HD is relatively robust to small head movements. However, increased infra-red ‘bounce’ was observed during the sessions with a chinrest, where small and temporary reflections from the chinrest would cause it to be mistakenly detected as a part of the eye. Improvements in tracking accuracy expected from use of the chinrest may have been lost as a result. While this problem is solvable by eliminating infra-red reflections, a possibility for development would be for Gazepoint to offer a user intervention enabling the operator to manually correct the misidentified reflections during calibration, such that the Gazepoint algorithm would subsequently underweight those regions when estimating gaze location. Similarly, providing the stream of estimated distances from the screen, in addition to the gaze coordinates and pupil samples, would be useful for researchers to accommodate changes in distance from the screen in non-chinrest conditions.

## Experiment 2

Experiment 1 provided a standard validation of the GP3-HD eye-tracker in terms of its accuracy and precision, degree of data loss and the benefits of head stabilization. While this provides a useful benchmark assessment of the GP3-HD, such eye-tracking validation studies do not reflect typical experiments in which task demands are usually greater and there are more limited checks on the eye-tracker’s performance imposed by study design (Niehorster et al., 2018). Therefore, in Experiment 2 we provide data from a real-world typical psychological experiment to assess GP3-HD performance. Importantly, as typical behavioral experiments may vary from a few minutes to hours, we investigated how data quality parameters changed over time, across a 1-h-long experiment.

Experiment 1 also demonstrated that GP3-HD is able to capture pupil changes such as the PLR. In most cognitive and behavioral research, however, researchers are typically interested in how pupil changes are modulated by cognitive and affective factors rather than low-level visual properties. This can range from quantifying cognitive effort (Papesh & Goldinger, 2012; Piquado et al., 2010) to emotional arousal (Bradley et al., 2008). Considering that cognitive and emotional effects on pupil diameter are much smaller in magnitude than luminance effects (Mathôt, 2018), it is unclear how well the GP3-HD is able to capture such effects. In Experiment 2 we used data from a paradigm that allowed us to explore how pupil size is modulated by emotional factors. Specifically, the effect of viewing emotionally arousing images on pupil diameter was investigated. We predicted a positive correlation between self-rated arousal and pupil size. Since this task makes use of naturalistic visual stimuli varying in low-level properties, the luminance of the stimuli can also be regressed onto pupil size to model modulation of pupil size due to the PLR. Stimulus brightness should have a negative correlation with pupil size, such that brighter stimuli should lead to decreased pupil size (pupil constriction) whereas darker stimuli should predict increased pupil size (pupil dilation).

Additionally, in Experiment 2, SC and HR data was collected from participants simultaneously using both the GPB and a well-validated physiological recording system (BIOPAC-MP160). Strong correlations between devices would indicate the capability of the GPB to capture physiological signals.

### Method

#### Participants

A total of 46 healthy participants with no recent history of mental health problems (28 female, 18 male, M = 23, SD = 5.54, 18 to 65 years old), with normal or corrected to normal vision and without makeup, took part in this study. Additionally, ten participants with an independent diagnosis of autism were recruited at a later stage and their data used for a subset of the heart rate analyses (four female, six male, M = 38, SD = 15.73, 18–65 years old). Participants took part in a psychological experiment where they had to view and rate emotional pictures, while their gaze and physiological signals were monitored. Participants were reimbursed £10 per hour or 3 course credits for their time.

#### Stimuli and task

**Fig. 6:**
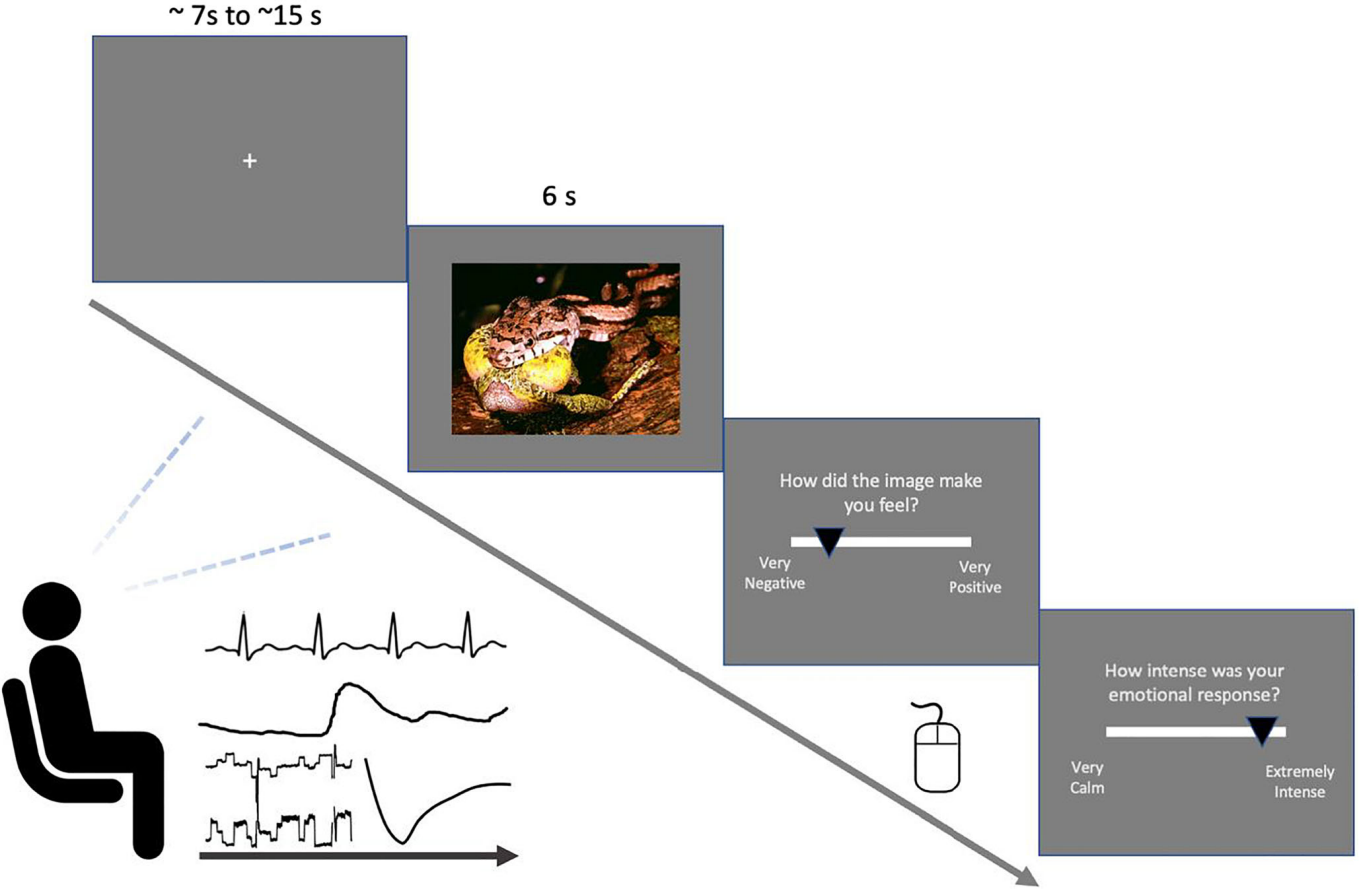
Schematics of Experiment 2

Picture stimuli were 50 images from the International Affective Picture System (IAPS, Lang et al., 2005) designed to elicit emotional responses in observers (e.g., open lung surgery, naked bodies, etc.). The stimuli were chosen to cover a wide range of valence (M = 5.07, SD = 1.92, range = 1.46 – 8.19 – on a 1 to 9 scale) and arousal (M = 4.66, SD = 1.19, range = 2.63 – 7.21) scores based on population norms. Participants viewed each stimulus and provided ratings for valence using a slider (‘How did the image make you feel’) from ‘–10 (Extremely negative)’ to ‘+10 (Extremely positive)’; and arousal (‘How intense was your emotional response’) from ‘–10 (Extremely calm and relaxed)’ to ‘+10 (Extremely intense)’. Each trial started with a fixation cross of variable duration (ranging from 7 to 15s), followed by presentation of the image for 6 s, after which participants provided their valence and arousal ratings (see Fig. 6).

#### Apparatus and task environment

The experiment was displayed on a computer monitor measuring 1920 x 1200 pixels with a fixed refresh rate of 60 Hz running on an Intel Core i9 Windows 10 computer (32GB RAM).

#### Eye tracking

The same GP3-HD eye tracker from Experiment 1 was used to track eye-movements and pupil size with a sampling rate of 150 Hz. Setup and calibration were similar to Experiment 1. Recalibration was repeated after 25 trials (approximately 20 min). Participants were asked to keep their head and body still and a chinrest was used. However, they could move their eyes freely to explore the images.

#### Accuracy and precision

Accuracy and precision were computed in a similar manner as in Experiment 1, however here computations were performed during the fixation screen that preceded each trial. The data were trimmed to 6 s and the first 250 ms after the start of fixation cross were removed. Outlier precision and accuracy values were removed as in Experiment 1.

#### Physiological recordings

Physiological recordings of SC, pulse and heartbeat data were obtained from two systems: the GPB and the BIOPAC M160 with EDA100C and ECG100C modules (details below).

##### Skin conductance – GPB

The GPB measures both heart rate and skin conductance via a sensor attached to two fingers (index and middle finger), without the need for extensive skin preparation or specialized electrodes. To record SC, the GPB uses an exosomatic recording method which applies direct current with a constant voltage source. The applied voltage is 5 V through high impedance voltage division. The conditioning method uses an analog low pass filtered at 10 Hz with a sampling frequency of 150 Hz. The sensors use gold-plated steel electrodes strapped to distal phalanges of the fingers.

##### Heart rate – GPB

The SCR system described above allows detection of heartbeats at the middle or index finger using a photoplethysmography (PPG) method where a light beam is transmitted to the tissue and heartbeats are detected via intensity modulation of the reflected light. Note that the standard GPB system reports only heart rate data, however, through the provided API it is possible to store the raw pulse data using a custom script. This data was only available for a third of participants. Heart rate is computed using a moving average with a window of three beats. For all measures, live monitoring was achieved via the Gazepoint control application.

##### Skin conductance – BIOPAC MP160

To validate the GPB, physiological data were also collected using a BIOPAC MP160 system sampling at 2000 Hz. SC data were recorded using the EDA100C module and TSR203 transducers. The skin where the GSR electrodes were placed was cleaned with water and dried with a cotton tissue. The EDA100C uses a constant voltage (0.5 V) technique to measure skin conductance. SC was collected via two electrodes that were placed on the inside of the left foot to measure GSR (see Fig. S6 in Supplementary materials). Foot (rather than hand) placement was used to avoid creating interference between the two devices. The foot and fingers have been shown to be the best locations to measure SC and provide largely similar results (van Dooren et al., 2012).

##### ECG - BIOPAC MP160

The ECG signal was recorded via the ECG100C electrocardiogram amplifier, which records electrical activity generated by the heart. Two electrodes were placed on participants (see Fig. S7 in Supplementary materials). One electrode was placed on the right collarbone and one on the lower left torso to measure HR. All sensors were well secured with surgical tape to prevent loss or disruption of signal. The skin where the ECG electrodes were placed was cleaned with Signagel Electrode Gel before attaching sensors.

### Procedure

Following informed consent and the opportunity to ask questions, physiological recordings were prepared. A 2-min rest period was allowed for recordings to stabilize before preparations continued. The task started with a calibration, followed by another 2-min rest period, during which participants focused on a fixation cross in the center of the screen, and five practice trials. After 25 trials, participants took a short break for recalibration. After the task they were debriefed and compensated. All research was conducted in accordance with the revised 2013 Declaration of Helsinki and was approved by the local Research Ethics Committee.

### Data pre-processing

#### Eye-tracking

Eye-tracking data were pre-processed following the same steps as in Experiment 1. For pupil analysis, pupil response was baseline corrected using an interval corresponding to 1 s before and 1 s after the stimulus, using the same method as in Experiment 1.

#### Skin conductance and heart rate

SC data from both the GPB and BIOPAC systems were analyzed using a continuous decomposition analysis (CDA) algorithm implemented in the open-source software Ledalab (Kaernbach, 2005). For analysis, data were first downsampled to 10 Hz and inspected for artifacts. Adaptive smoothing was used prior to analysis. All optimizations used the default values in Ledalab recommended for SC measurement and analysis (Boucsein et al., 2012). The global mean of the SC signal as well as the event-related phasic signal was computed.

#### Heart rate data

**Fig. 7:**
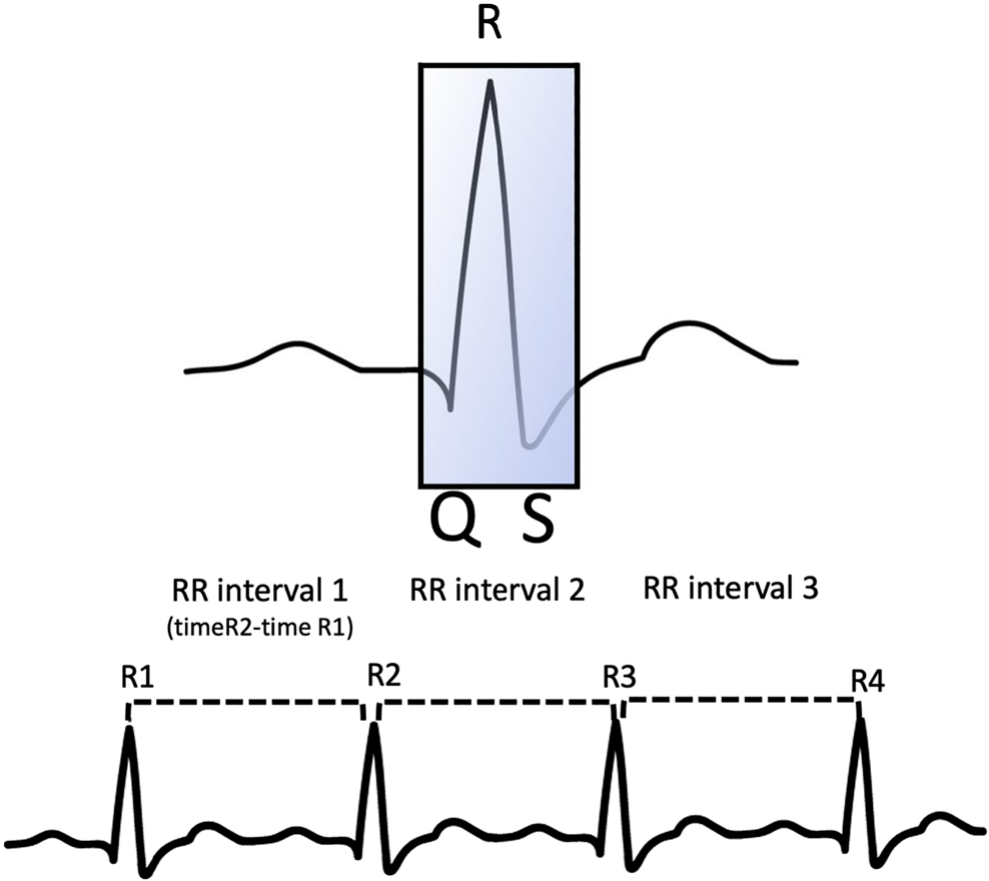
Illustration of the elements of a heartbeat used to compute heart rate and heart rate variability. RR-interval (or inter-beat interval - IBI) is the time difference between successive heartbeats. NN-interval is a normalized RR-interval in which artefacts have been removed

Established HR data-processing pipelines were used to process the heartbeat and ECG signal from the GPB and BIOPAC, respectively. Processing was accomplished via the ArtiiFact toolbox (Kaufmann et al., 2011). Artifact detection was achieved using the Berntson, Quigley, Jang, and Boysen (1990) algorithm based on individual thresholds derived from inter-beat-interval (IBI, also known as RR interval) distributions and their estimated real (not contaminated) distribution and interpolated using the cubic spline method. The peak of the R wave (heartbeat, see Fig. 7) was detected using the global threshold method (or local threshold method when drift was present), after low pass filtering at between 10 and 20 Hz.

In addition to HR, which was computed for the entire task, both time and frequency domain metrics of heart rate variability (HRV) were derived from the resting period. Here we report the standard deviation of NN intervals (SDNN), root mean square of successive RR interval differences (RMSDD), and percentage of successive RR intervals that differ by more than 50 ms (NN50) for the time domain; and absolute power of the high-frequency band (0.15–0.4 Hz) – HF, and low-frequency band (0.04–0.15 Hz) – LF for the frequency domain (Shaffer & Ginsberg, 2017).

### Statistical analyses

#### Eye-tracking

In addition to descriptive metrics for all gaze position and calibration measures, variance in data loss, accuracy and precision during the task is also reported. To this end, linear mixed models implemented in R package lme4 (Bates et al., 2014), were fitted to assess the rate of change over time. For example, the rate of data loss over the course of the experiment was investigated by modelling each of the 25 trials immediately following successful calibration for a total of 50 trials. Linear and quadratic functions were used to approximate the rate of change over time as these could easily describe linear or accelerated/delayed changes in the metrics. This approach is known as polynomial modelling or growth curve analysis (see, Mirman, 2017). Fitted models had a quasi-maximal structure (e.g., loss ~ (linear + quadratic) * block + (1 + linear + quadratic | participant id), with linear and quadratic representing orthogonal polynomial terms created as powers of the trial number, and trial id and participant id were estimated as a random intercept. Block was dropped as a random slope due to near zero variance and convergence errors.

#### Pupil size

Baseline-corrected pupil responses to each picture were regressed onto self-reported ratings of arousal and mean brightness values for each image, while controlling for random effects of participant and stimulus id. Random slopes were dropped due to near zero variance or convergence errors, and the final models had the form: pupil ~ arousal rating + mean brightness + (1 | stimulus id) + (1 | participant id). All continuous predictors were mean-centered and scaled.

#### Skin conductance and heart rate

The average SC signal during each trial, event-related phasic SC responses and HR metrics were correlated between the GPB and BIOPAC systems. HRV measures were only computed for the subsample of participants for whom the raw timeseries of ECG/Pulse data was available for both the GPB and BIOPAC. This subsample had 20 participants with raw pulse measurements (ten neurotypical individuals included in the other analyses, and an additional ten autistic participants). For this subsample, all correlations are Kendal rank correlations due to small sample sizes.

### Results

#### Eye-tracking

##### Calibration

Calibration quality and other descriptive statistics are provided in Table 4. The average calibration error across all three accepted calibrations during the 1-h session was within the expected range, with a mean of .98° (SD = .45°), and it took an average of 1.66 attempts to achieve an acceptable calibration.

**Table 4:** Descriptive measures for eye tracking data

Variable	Mean	SD	Range
Calibration count	1.66	0.98	1–5
Calibration error	0.98	0.45	0.49–4.92
Gaze loss	8.3%	15%	0–100
Accuracy			
Global	0.77	0.70	0.23–13.53
Vertical	1.33	0.73	0.27–9.50
Horizontal	0.54	0.50	0.30–6.34
Precision			
Global	0.27	0.11	0.15–2.14
Vertical	0.30	0.13	0.11–.95
Horizontal	0.24	0.09	0.09–.60

*Notes*. Calibration error, accuracy, and precision data is reported in degrees of visual angle (range is reported at the trial level)

##### Data loss

On average, less than 10% of gaze data were lost, which is consistent with the typical rate of loss reported in the literature and even lower than some more expensive systems (e.g., Tobi TX300 based on (Holmqvist, 2017). During the task less than 1% of the data fell outside screen bounds.

Data loss increased at a linear rate (estimate = .004, SE – .002, *t* = 2.05, *p* = .04) across the experiment. There was also a main effect of block, with trials after the recalibration showing reduced data loss (estimate = –.12, SE = .02, *t* = – 5.08, *p* < .001). The only significant interaction was between the quadratic term and block (estimate = .05, SE = .02, *t* = 2.43, *p* = .02): post hoc tests indicated that the rate of loss in the first block additionally followed an ‘inverted-U’ shape, that is, it was characterized by rapid increase in data loss in the first few trials until the rate of loss stabilized and reduced in the last few trials (estimate = .01, SE = .002, *t* = 5.07, *p* < .001).

#### Accuracy

**Fig. 8:**
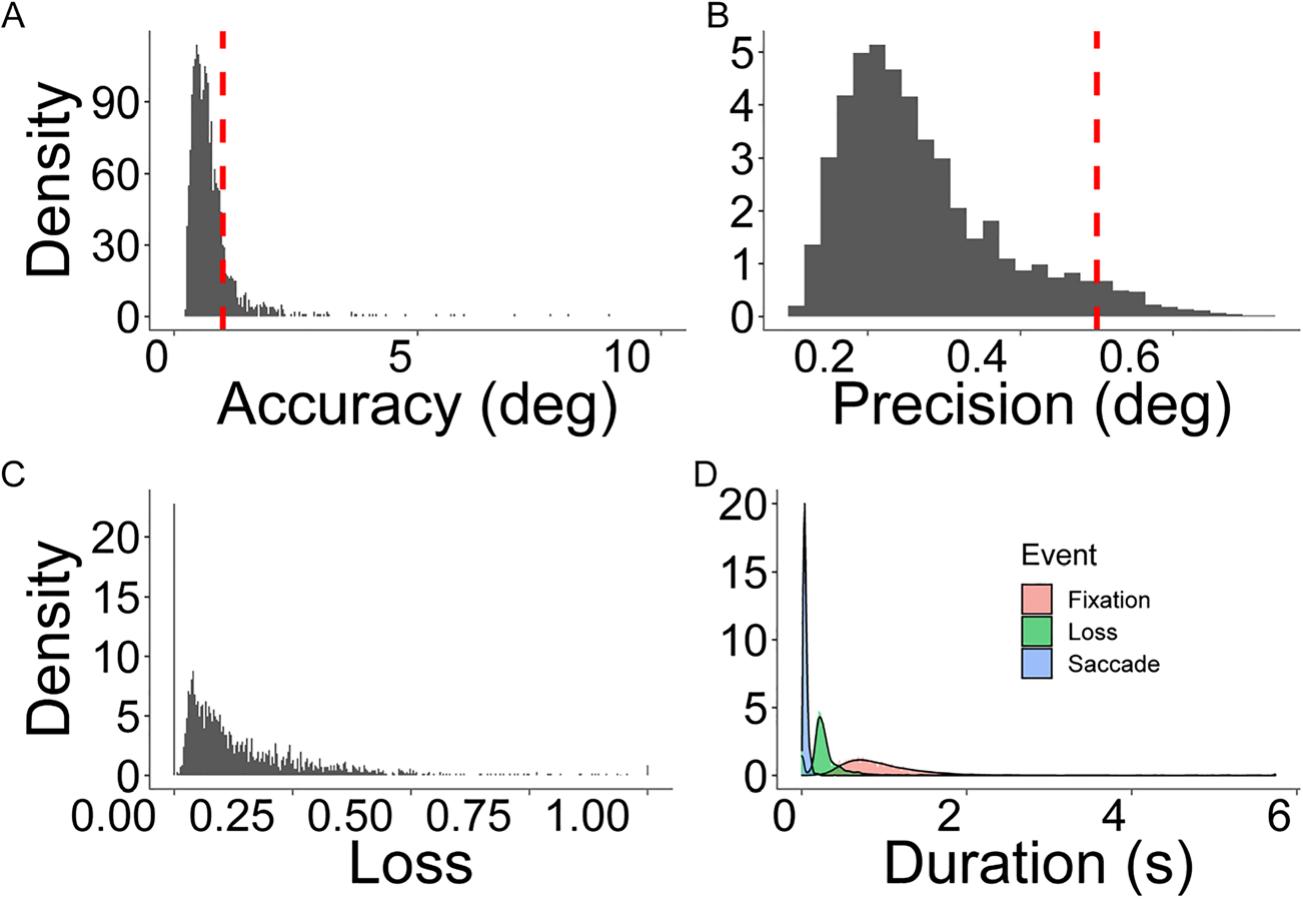
Density plots for accuracy (**a**), precision (**b**), and data loss (**c**). Frequency of fixations, saccades, and data loss for each trial and participant (**d**). Data represents the density of every single observation for every participant for each trial

Accuracy values were in the expected range, averaging between 0.5° and 1.33°, with better accuracy on the horizontal dimension with values consistently ~ 0.5° (see Fig. 8). Overall, the global accuracy changed at a linear rate such that for every trial, tracking accuracy deteriorated by 0.04° (estimate = .04, SE = .01, *t* = 3.21, *p* = .001). There were also block differences such that the second block showed better accuracy, with re-calibration improving accuracy by .06° compared to the previous trial (estimate = .06, SE = .02, *t* = 2.17, *p* = .01). Results were consistent for vertical and horizontal accuracy.

With respect to horizontal accuracy, for every trial after calibration tracking accuracy deteriorated by .03° (estimate = .03, SE = .009, *t* = 3.21, *p* = .001) and recalibration after every 25 trials improved accuracy by on average .05° (estimate = .05, SE = .02, *t* = 2.49, *p* = .01). For vertical accuracy there was an interaction between block and the linear parameter (estimate = .05, SE = .02, *t* = 2.79, *p* < .001), such that in the first block accuracy declined more linearly than in the second block.

#### Precision

All precision values were within an acceptable range < 0.5°, with the horizontal dimensions showing better tracking precision. There was no effect of trial on precision nor interactions with block. There was only a main effect of block, with the second block showing better precision (estimate = .007, SE = .002, *t* = 2.93, *p =* .002). Both vertical and horizontal precision showed the same effect.

#### Pupil size

**Fig. 9:**
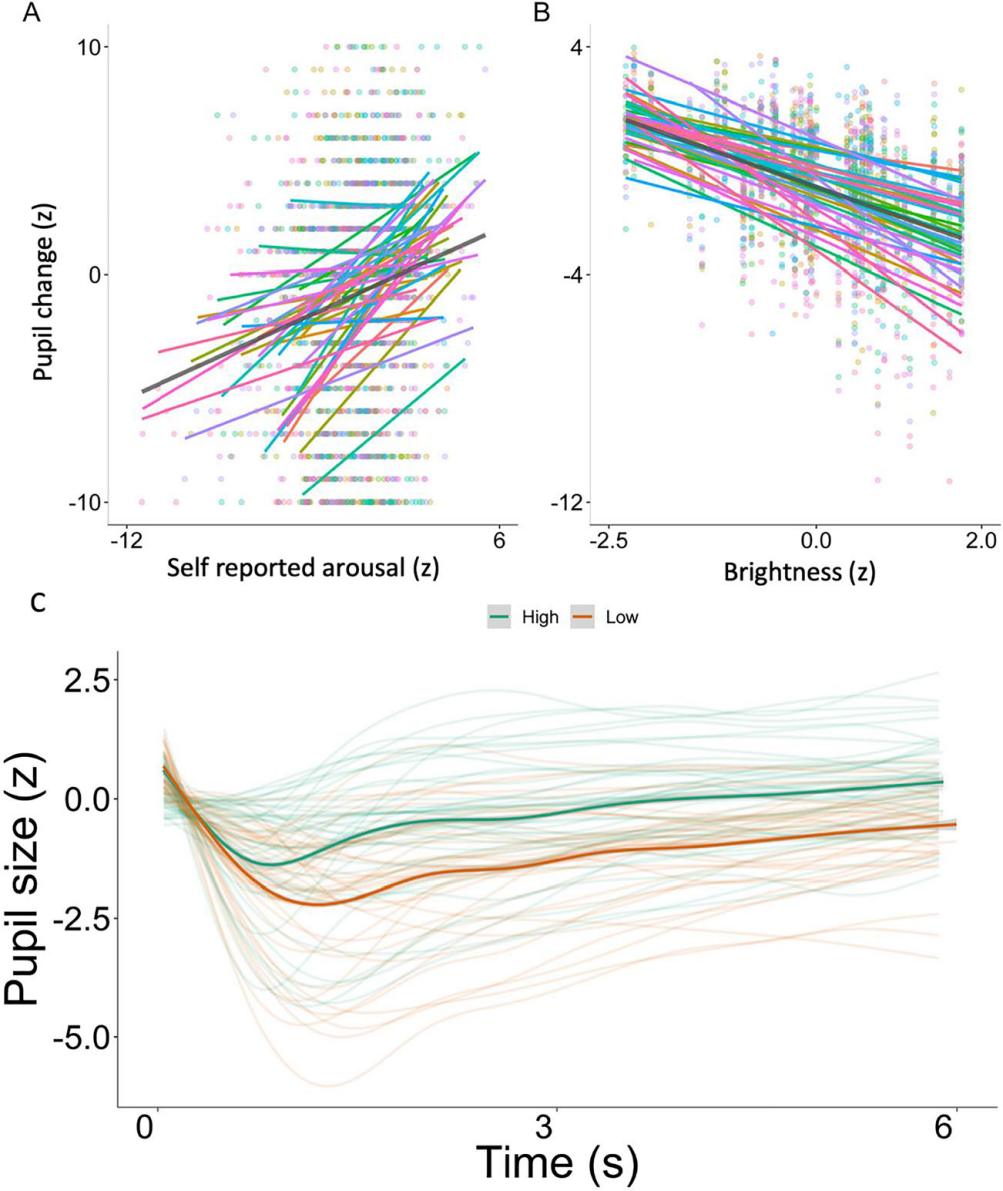
Self-perceived arousal in response to emotional stimuli correlated with pupil size (**a**) as did stimulus luminance (**b**). **c** The time course of pupil response by high and low arousal stimuli (split for visualization only). Individual lines represent individual participants average pupil trace

Experiment 2 aimed to explore whether the modulation of pupil size by emotional arousal is detectable with the GP3-HD, considering that such effects are orders of magnitude smaller than the PLR detected in Experiment 1. A main effect was observed for self-reported arousal in the predicted direction (estimate = .24, SE = .05, *t* = 4.35, *p* < .001), which remained significant after statistically controlling for brightness. This shows that self-perceived arousal is positively related to pupil change. The predicted effect of brightness on pupil size was also significant, and larger (estimate = – 1.02, SE = .12, *t* = – 8.59, *p* < .001), demonstrating again that brightness is negatively related to pupil change. As predicted, the effect of arousal on pupil size was less than 1% of the size of the brightness effect and showed more individual variability in comparison to the PLR (see Fig. 9).

The model explained 54% of the variance in pupil size, of which 23% was explained by the fixed effects (discounting the random effects of participants and trial). Thus, Experiment 2 provides support for the use of the GP3-HD eye tracking system for the study of gaze position and pupil size in typical psychological experiments. Tracking capability is better horizontally than vertically, but the vertical accuracy also remained acceptable range (~ 1^°^).

**Fig. 10:**
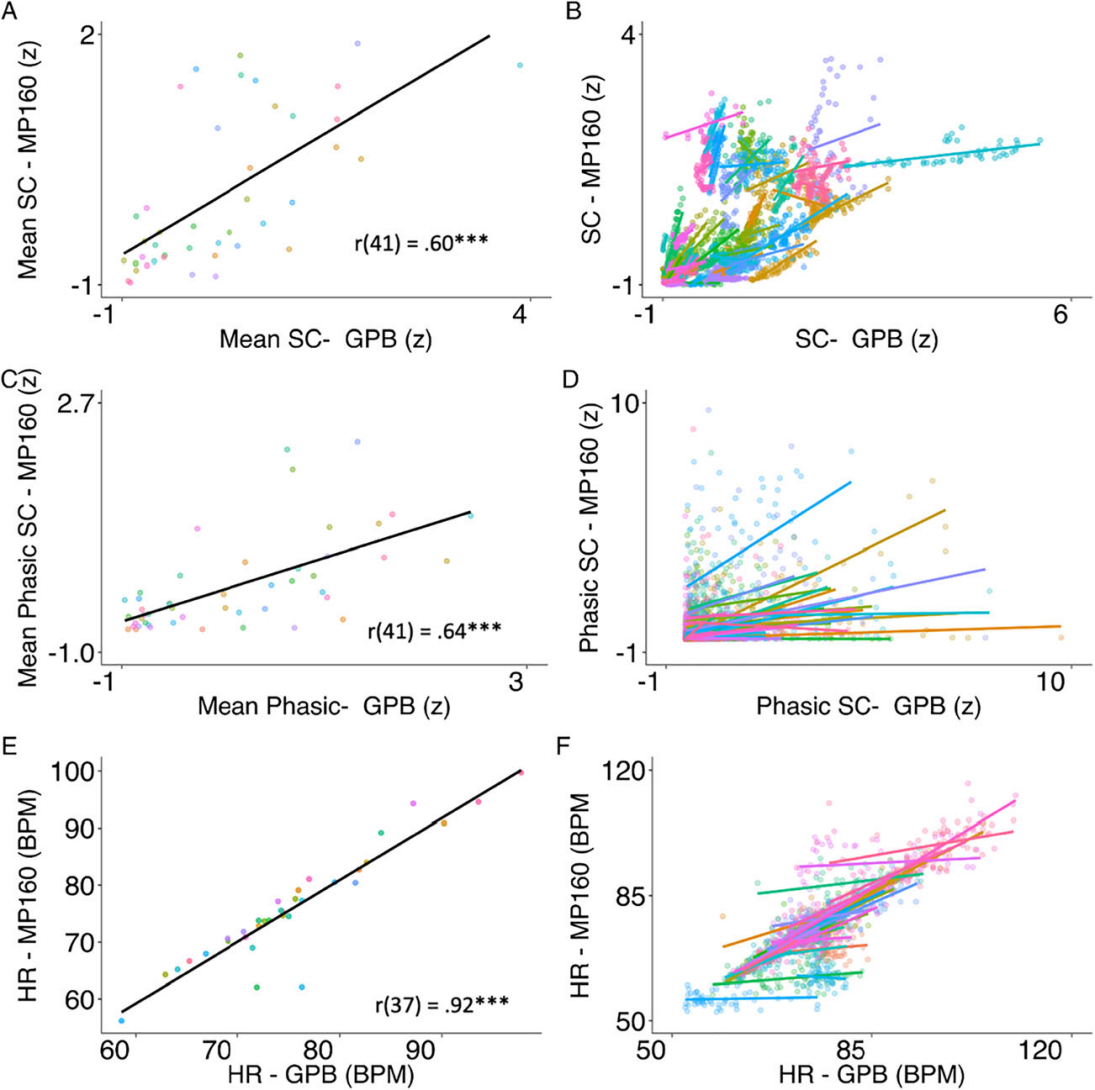
**a**–**d** Scatter plots for raw averages of the skin conductance signal across the Gazepoint biometrics (GPB) and BIOPAC MP160 systems. **e**–**f** Scatter plots for correlations of computed heart rate from Gazepoint biometrics system and BIOPAC MP160 ECG. *Left plots* show data aggregated by participant, and *right plots* show data for all trials and participants. Each *color* and *line* represent a single participant, each *dot* represents a single trial. *** *p* < .001

A second goal of this study was to provide a validation of the GPB system, by comparing SC and HR obtained using the GPB with data collected from the well-validated BIOPAC MP160 (see Fig. 10).

#### Skin conductance

The SC measurement capacity of the GPB showed excellent robustness, with less than .06% of loss compared to no loss of signal in the BIOPAC-MP160. The average SC signal from the GPB and BIOPAC showed a strong correlation (*r*_(37)_ = .60, *p* < .001). This was also consistent when looking at specific derived measures of skin conductance from Ledalab, such as a decomposed phasic signal (*r*_(41)_ = .64, *p* < .001), with measurements across both devices sharing 40% of the variance. However, as illustrated in Fig. 10, there is a significant degree of difference in how well these signals correlate at an individual level. This may be related to differences in how similar the physiological properties are in the measured locations for each participant (foot vs. palm), as well as the fact that the SC signal from the GPB is smaller in range compared to the BIOPAC signal.

A sample of the SC signal from an example participant is shown in Fig. S5 in the Supplementary materials for both the GPB and BIOPAC systems. One obvious difference is that the signal from the BIOPAC is much larger in magnitude compared to the GPB signal. This results in more signal being preserved after removal of tonic data in BIOPAC compared to GPB data.

#### Heart rate

Quality checks indicated that the GPB lost on average 19% (SD = .37) of data (impossible HR values or loss of signal) compared to no obvious data loss for the BIOPAC system (i.e., peaks were still present even in periods of relative noise). Importantly, however, participants were not more likely to lose data as the task progressed (estimate = .004. SE = .005, *t* = .69, *p* = .45). This was a concern as the strap of the sensors may be thought to limit the blood flow to the finger, systematically affecting heartbeat tracking over time. HR recorded from the GPB and BIOPAC were, however, very strongly correlated (*r*_(37)_ = .92, *p* < .001, Fig. 10), demonstrating that the GPB provides valid measurement of heart rate, corresponding to systems which are much more expensive. Results were similar whether using interpolated or non-interpolated GPB heart rate data. Notably, these correlations are much larger than the correlations between the systems for SC signals, however the loss of HR data is much greater than the loss of SC signals for the GPB system.

Another ECG metric of interest for many researchers is heart rate variability (HRV). Notably, HRV measures require more sensitive and less noisy recordings than HR, which is relatively stable.

#### Heart rate variability

Both time and frequency domain measures of HRV were considered. Overall, there were strong correlations between GPB and BIOPAC MP160 recordings and derived HRV metrics. All correlations were > .6 (see Table 5).

**Table 5:** Heart rate and heart rate variability correlations between the GBP and BIOPAC systems

Variable	Correlation
HR	.92^***^
Time domain	
SDNN	.73^***^
PNN50	.63^***^
RMSSD	.61^***^
NN50	.65^***^
Frequency domain	
HF	.75^***^
LF	.76^***^

*Notes*. *N* = 20. Analyses are reported excluding outliers, but results did not differ significantly with the inclusion of outliers (there was only one outlier per analysis). *** *p* < .001. All correlations are Kendal rank correlations. *HR:* Heart rate; *SDNN:* Standard deviation of NN intervals; *RMSSD:* Root mean square of successive RR interval differences; *NN50:* Percentage of successive RR intervals that differ by more than 50 ms; *HF:* Absolute power of the high-frequency band (0.15–0.4 Hz); *LF:* Low-frequency band (0.04–0.15 Hz)

**Fig. 11:**
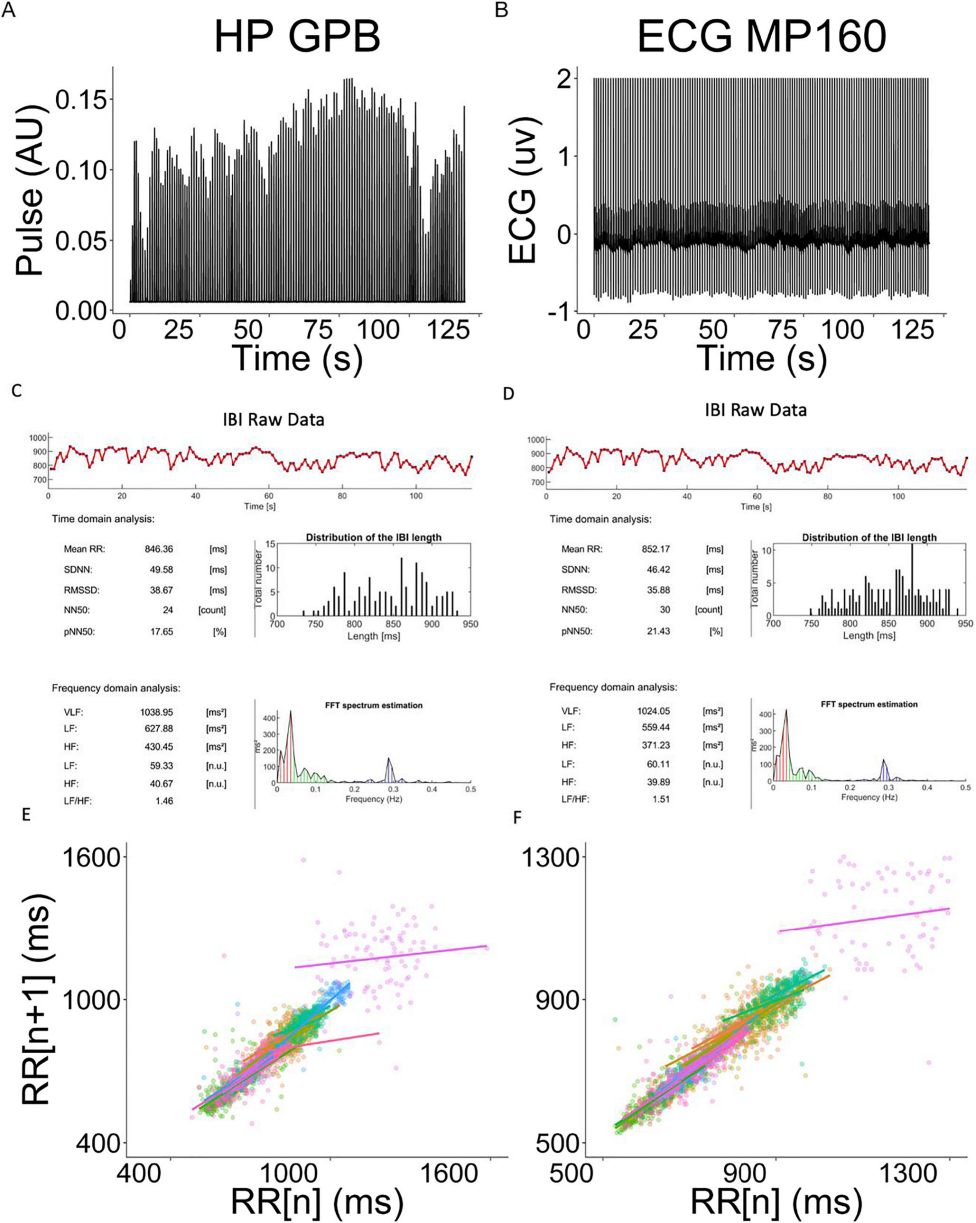
Comparison plots of heartbeat data from the Gazepoint biometrics (GBP) and BIOPAC MP160 systems. **a** and **b** show a representative participant’s raw recording of pulse (GPB; A) and electrocardiogram (BIOPAC MP160; B) data. **c** and **d** show derived heart rate variability metrics for the same participant across the two devices (C = GPB, D = BIOPAC). **e** and **f** show point-care plots for the inter-beat-interval (IBI) for all participants. Each *color* denotes a single participant, each *dot* denotes a single RR (peak-to-peak) duration

Overall, the GPB system shows strong concordance with the well-established BIOPAC MP160 system. Notably, the HR measurements were much more consistent than SC, despite the increased data loss (see Fig. 11).

### Discussion

Experiment 2 provided further validation of the accuracy, precision, and robustness of the GP3-HD, with accuracy generally within 1° of visual angle and precision < 0.5°. In addition, correspondence was observed between self-reported arousal and pupil size, showing that the small changes in pupil size due to emotional arousal can be measured using the GP3-HD. Furthermore, the PLR was measured in response to luminance changes which were less marked than those in Experiment 1. The changes over time observed for data loss, accuracy and precision are consistent with observations that eye-tracking data quality deteriorates over time (Holmqvist et al., 2012; Hessels et al., 2017). Researchers should accommodate this in study design, for example by including frequent recalibration or breaks to reduce participants’ fatigue.

Finally, the comparison between SCR and HR raw and derived metrics suggests moderate to very strong agreement between the GPB and the well-established BIOPAC MP160 system, with correlations ranging from .6 to .95. However, the low amplitude of the SC signal from the GPB system means it is likely to make the separation of tonic and phasic components more difficult (Edelberg, 1993; Society for Psychophysiological Research Ad, 2012), than the SC signal from the BIOPAC system.

Nonetheless, estimation of resting and stimulus-evoked SC responses is possible, as indicated by a relatively strong correlation of SC measurements between devices. For both heart rate and heart rate variability metrics (in both the frequency and time domain) the recordings from the GPB showed remarkable consistency with those from the BIOPAC system. However, measurement of the pulse by the GPB system was more prone to data loss than the ECG system used by the BIOPAC. This data loss may cause problems for heart rate variability analyses.

## General discussion

Experiments 1 and 2 aimed to assess the validity of the GP3-HD eye-tracking system. While the manufacturer’s stated levels of accuracy are possible to achieve (0.5–1°), in two studies the average accuracy of the system was closer to 1°, with the horizontal accuracy and precision matching the stated ~ 0.5 and < 0.5, respectively. This is similar to what has been reported for similar grade (e.g., EyeTribe, Tobii EyeX) as well as higher grade devices (e.g., Tobii TX 300) in large-scale eye-tracking comparison studies, where the measured accuracy averaged around 1^o^ (Funke et al., 2016; Holmqvist, 2017). Data loss is also a good indicator of the capabilities of a system and in this regard, the GP3-HD also performs quite well, with discarded data after cleaning making up on average less than 10% of the collected data, compared to reported data loss in different systems which ranges from less than 2% to up to 20% (Holmqvist, 2017).

It is important to note that in behavioral studies, reduced accuracy can also result from participant behavior rather than hardware limitations, as the computation of accuracy and precision is reliant on participants attending to the targets. Nonetheless, this type of validation represents the most likely scenario under which most eye-tracking systems will be used with human participants. The lack of any major effects on the quality of gaze estimation between chinrest and no-chinrest conditions in Experiment 1 suggests that the gaze estimation algorithm for the GP3-HD is robust. It is worth noting, however, that in both conditions participants were instructed to avoid body and head movements. Other studies have shown that body and head position can severely affect the quality of data obtained from remote eye-trackers (Niehorster et al., 2018), and that infants and participants with neuropsychiatric conditions are more likely to show poor data quality, in terms of calibration, accuracy, precision and data loss (Dalrymple et al., 2018; Hessels & Hooge, 2019; Holmqvist et al., 2012). Therefore, we recommend that experimenters systematically assess data quality parameters when using GP3-HD for experimental research.

One issue to consider when using the GP3-HD is that tracking participants’ gaze when using chinrests or with additional objects near their head (e.g., headphones, glasses, masks) may cause infrared bounce. We observed this during some recordings, where transient reflections from headphones (which was required for a separate task) or the chinrest would cause gaze estimation failures. While these data samples are typically flagged as invalid by the GP3-HD algorithm, it can increase data loss in some cases sufficiently to invalidate a full trial if no correction is made. Simple solutions, such as covering areas that are likely to be reflective with non-reflective tape, are usually enough to solve this issue.

Analyses of gaze position metrics show that detection of fixations and saccades is reliable, yet measurement of the kinematics of saccades are negatively impacted by the low sampling rate. Similarly, analyses of velocity profiles show that known relationships between saccade parameters, e.g., saccade velocity and amplitude, or saccade duration and amplitude, recorded from the GP3-HD only approximate the expected relationships. Nonetheless, any inaccuracy in the estimation of fixation and saccade metrics is likely systematic, such that comparisons between different conditions, individuals or groups should be possible, if all other factors are taken into account (e.g., differences in accuracy or precision).

Finally, in terms of the software for collection and analysis of data, the GP3-HD software is unlikely to meet the demands of most experimental research. However, using the Gazepoint API, a number of popular experimental software libraries now support Gazepoint eye-trackers, such as PsychoPy (Peirce et al., 2019), PyGaze (Dalmaijer et al., 2014), OpenSesame (Mathôt et al., 2012), and Psychtoolbox (Kleiner et al., 2007). Similarly, for analyses, the output generated by Gazepoint can be imported into third party open-source software like Python and R (or proprietary software such as MATLAB) for further processing.

### Gazepoint biometrics system

Overall, the GBP system showed a high degree of consistency with the well-established (and considerably more expensive) BIOPAC system, which is often considered to be the ‘gold-standard’ for physiological recording. However, specialized pre-processing of pulse data obtained from the GBP system is necessary for calculation of HRV metrics. Similarly, like other PPG measures, the study of properties of the pulse (or heartbeat) signal, such the QRS complex, is likely to be challenging (although see Chiu et al., 2020). While the SC signal is more robust to data loss, the low amplitude of the signal is likely to make the separation of phasic and tonic measures of SC more difficult (Edelberg, 1993; Society for Psychophysiological Research Ad, 2012). Another problem relates to motion and respiration artifacts. Irregular respiration and deep breaths cause fluctuations in the SCR signal, which may lead to inaccurate SCR detection (Posada-Quintero & Chon, 2020). In systems like the BIOPAC MP160, it is possible to also collect respiration (with additional modules) and using this information to remove artefactual SCRs caused by respiration. Such automation is impossible with the GPB. This also means that tasks involving physical activity cannot be used when measuring SC and HR with the GPB. The GPB design is also optimized for use in the right hand, while it works on the left hand the positioning is less ideal, which may be a problem in reaction-time tasks where the use of a dominant right-hand is needed.

In terms of software integration, at the time of writing, raw recordings of SC, HR, and pulse are not accessible by default in the implementation of Gazepoint systems in PsychoPy, OpenSesame or Psychtoolbox. However, it is possible to access these data through the provided Gazepoint API. Similarly, the current iteration of Gazepoint’s collection and analysis software does not include the raw pulse rate, although it is likely to be included in future releases. As with the eye-tracking data, however, output from the GPB can be exported to be used in open-source toolboxes for analyses of SC such as Ledalab (Kaernbach, 2005) or PSPM (Bach & Staib, 2015), or heart rate variability such as Artifact (Kaufmann et al., 2011) or Kubios (Tarvainen et al., 2014). Based on our tests, simple k-means clustering on the raw timeseries of pulse data from the GPB provided acceptable classification of heart beats, which means that basic processing pipelines can be used relatively easily.

## Conclusions

Two experiments assess the validity of a new relatively low-cost eye-tracking and psychophysiology system from Gazepoint. We show that the GP3-HD eye-tracker shows acceptable accuracy and precision, with only the study of saccade kinematics likely to be problematic. The GP3-HD was also shown to reliably capture the PLR and arousal effects on pupil size. Measurement of SC, HR and HRV from the GPB show a high degree of consistency with the well-established BIOPAC MP160 system. However, the low amplitude of SC signal may make it difficult to parse small phasic responses, and the relatively high degree of pulse rate loss in some participants may render pulse data unsuitable for HRV analyses without extensive pre-processing.

## Supplementary Information

https://osf.io/8qxk4/

ESM 1 (DOCX 4803 kb)

## Footnotes

1 Note that although eye-tracking is also considered to be a psychophysiology technique, we use the terms separately in this paper simply to aid exposition of the validation of the eye-tracking device and the biometrics bundle (for skin conductance and heart rate).

2 Please note that the screen was not exactly white as the change from a completely black to a completely white screen would have been straining for participants and would have caused excessive blinks and data loss during this transition period. The exact color of the screen was [0, 0, 0] in the RGB color space.

3 Average error of the accepted calibration is measured in degrees of visual angle.

## Declarations

All research procedures were in accordance with the revised 2013 Declaration of Helsinki.
