# Supplementary material for: Validation of Gazepoint low-cost eye-tracking and psychophysiology bundle
Source: Behav Res Methods. 2021 Aug 17;54(2):1027–49. doi: 10.3758/s13428-021-01654-x (PMC9046335; doi:10.3758/s13428-021-01654-x)
Supplement: Supplementary file 1 — (DOCX 4803 kb) [file 13428_2021_1654_MOESM1_ESM.docx]

**Supplementary materials**

**Accuracy analyses with outliers**

**Table S1.**

*Average vertical, horizontal and global accuracy without exclusion of outlier samples*

|  | Accuracy | | |
| --- | --- | --- | --- |
| Target dot | Vertical | Horizontal | Global |
| *Chinrest* |  |  |  |
| Central | 1.76 | 0.85 | 2.03 |
| Left | 1.03 | 1.05 | 1.58 |
| Lower central | 1.87 | 0.78 | 1.97 |
| Lower left | 1.62 | 1.56 | 2.33 |
| Lower right | 1.86 | 1.97 | 2.79 |
| Right | 1.10 | 1.63 | 2.07 |
| Upper central | 1.79 | 0.50 | 1.84 |
| Upper left | 1.08 | 0.86 | 1.43 |
| Upper right | 1.40 | 1.17 | 1.92 |
| *No chinrest* |  |  |  |
| Central | 1.85 | 0.74 | 2.04 |
| Left | 1.01 | 1.23 | 1.74 |
| Lower central | 1.85 | 0.55 | 1.94 |
| Lower left | 1.67 | 1.38 | 2.27 |
| Lower right | 1.55 | 1.63 | 2.31 |
| Right | 1.07 | 1.30 | 1.79 |
| Upper central | 1.83 | 0.46 | 1.85 |
| Upper left | 1.17 | 1.08 | 1.65 |
| Upper right | 1.28 | 0.98 | 1.67 |

*Notes.* Accuracy is reported in degrees of visual angle. The higher the value, the higher the error between the estimated gaze location and the location of a known target (~ the lower the accuracy).

**Table S2.**

*Linear mixed models with two fixed factors (condition and dot position) and vertical, horizontal, and global accuracy as dependent variables*

| Model equation: **Vertical accuracy** ~ Dot position + Condition + (1 \| Participant) + (1 \| Trial) | | | | |
| --- | --- | --- | --- | --- |
|  | Estimate | SE | t | p |
| **Intercept** | 0.99 | 0.05 | 21.94 | .000 |
| **Dot position (Peripheral – Central)** | -0.15 | 0.03 | -4.69 | .000 |
| Condition (No chinrest – Chinrest) | 0.02 | 0.01 | 1.19 | .234 |
| Model equation: **Horizontal accuracy** ~ Dot position + Condition + (1 \| Participant) + (1 \| Trial) | | | | |
|  | Estimate | SE | t | p |
| **Intercept** | 0.54 | 0.05 | 11.29 | .000 |
| **Dot position (Peripheral – Central)** | 0.16 | 0.04 | 4.01 | .000 |
| Condition (No chinrest – Chinrest) | -0.03 | 0.02 | -1.79 | .075 |
| Model equation: **Global accuracy** ~ Dot position + Condition + (1 \| Participant) + (1 \| Trial) | | | | |
|  | Estimate | SE | t | p |
| **Intercept** | 1.08 | 0.04 | 26.96 | .000 |
| Dot position (Peripheral – Central) | -0.05 | 0.02 | -2.03 | .051 |
| Condition (No chinrest – Chinrest) | -0.0004 | 0.01 | -0.03 | .977 |

*Notes.* No samples were excluded for these analyses. The higher the value, the higher the error between the estimated gaze location and the location of a known target (~ the lower the accuracy).

**Bayesian analyses for condition comparisons**

All Bayesian analyses used default priors in JASP.

**Calibration quality**

**DV = Number of calibration attempts**

| **Table S3.**  *Bayesian Wilcoxon Signed-Rank Test* | | | | | | | | | | | |
| --- | --- | --- | --- | --- | --- | --- | --- | --- | --- | --- | --- |
| **Measure 1** | |  | | **Measure 2** | | **BF₁₀** | | **W** | | **Rhat** | |
| Chinrest |  | - |  | No chinrest |  | 0.326 |  | 75.000 |  | 1.000 |  |
|  | | | | | | | | | | | |
| *Note.* Result based on data augmentation algorithm with 5 chains of 1000 iterations. | | | | | | | | | | | |

| 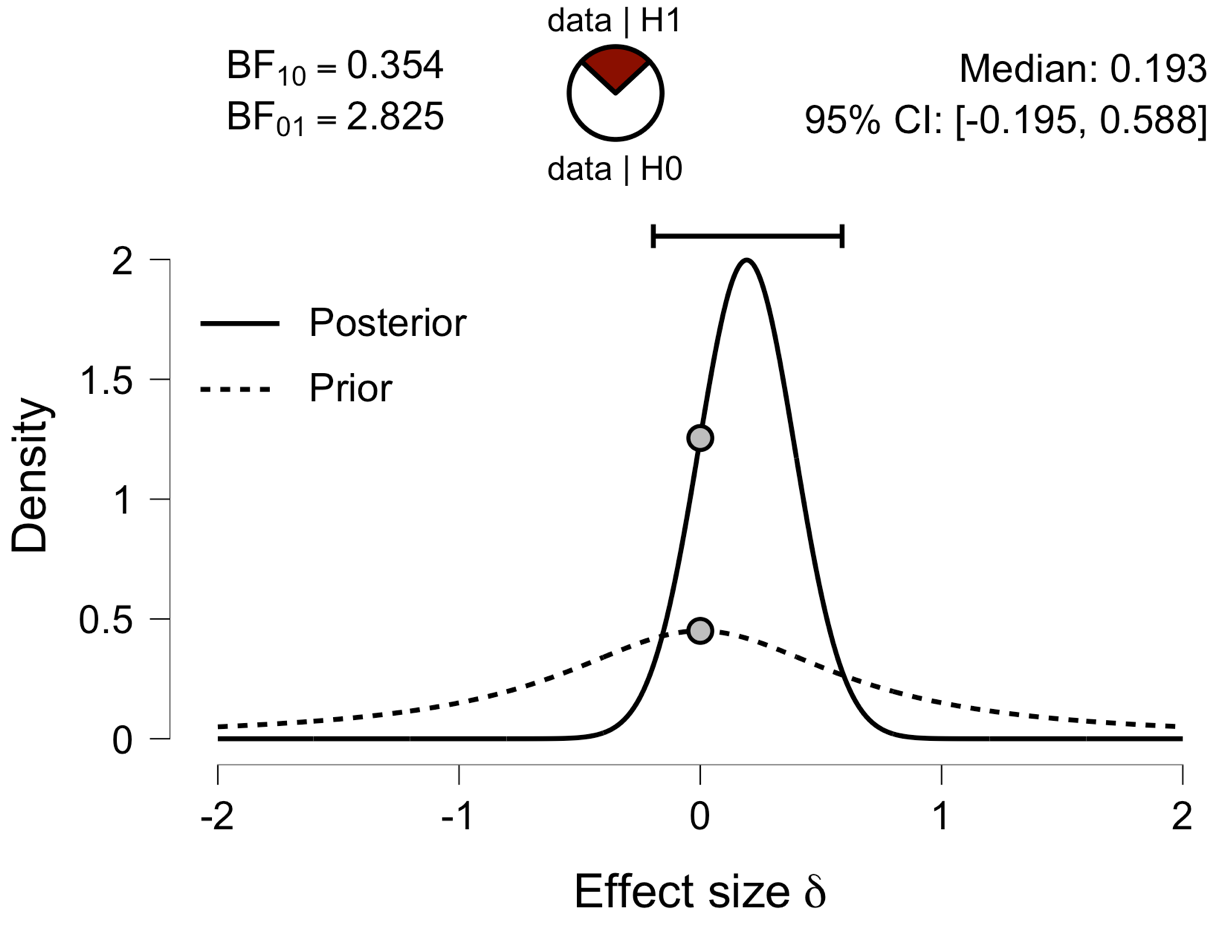  *Figure S1.* Inferential plots  **DV = Average error of the accepted calibration**  **Table S4.** | | | | | | | | | | | |
| --- | --- | --- | --- | --- | --- | --- | --- | --- | --- | --- | --- |
| *Bayesian Wilcoxon Signed-Rank Test* | | | | | | | | | | | |
| **Measure 1** | |  | | **Measure 2** | | **BF₁₀** | | **W** | | **Rhat** | |
| Chinrest |  | - |  | No chinrest |  | 0.218 |  | 178.000 |  | 1.000 |  |
|  | | | | | | | | | | | |
| *Note.* Result based on data augmentation algorithm with 5 chains of 1000 iterations. | | | | | | | | | | | |


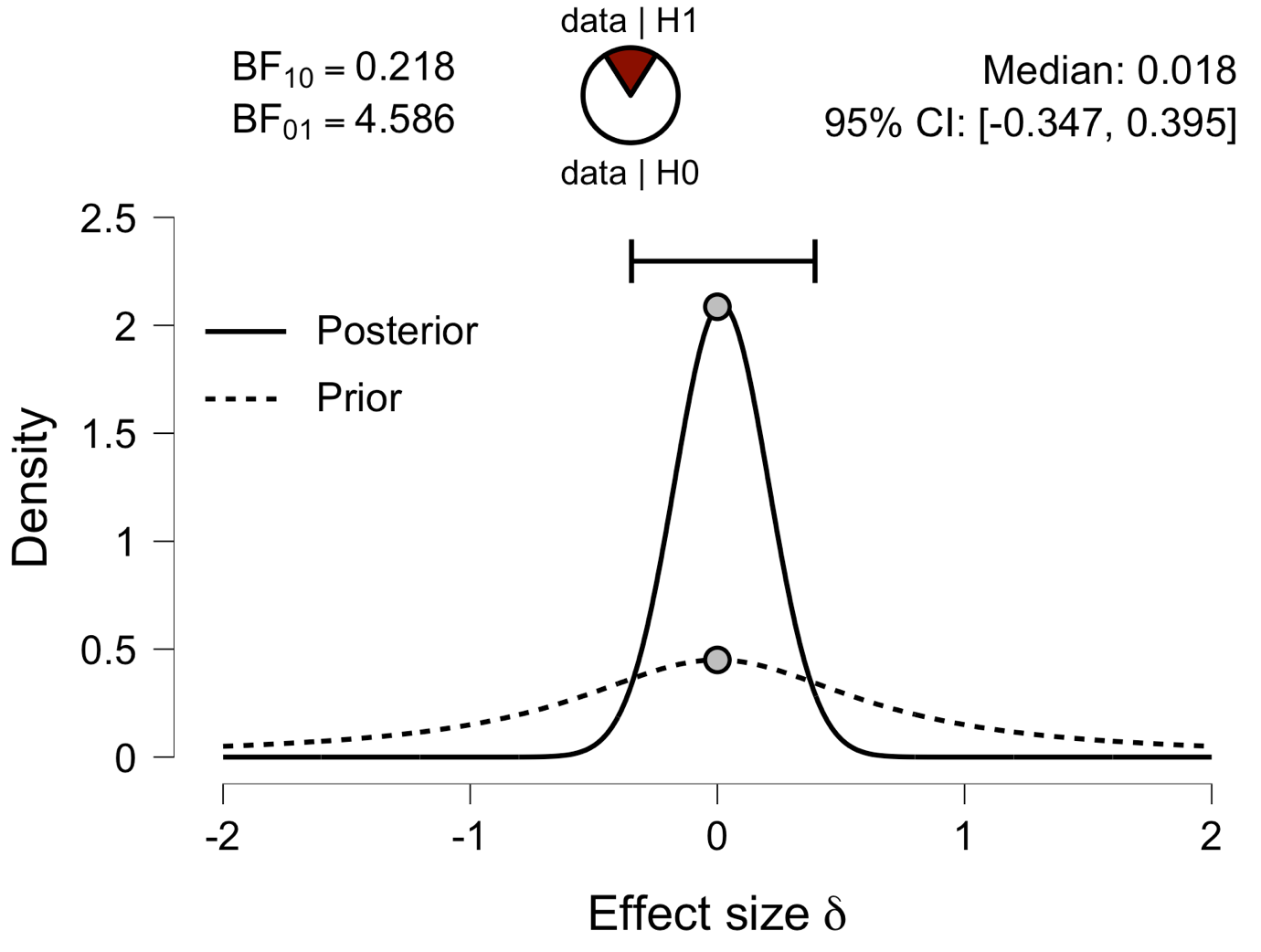


*Figure S2.* Inferential plots

**DV = Data loss**

**Table S5.**

| *Bayesian Wilcoxon Signed-Rank Test* | | | | | | | | | | | |
| --- | --- | --- | --- | --- | --- | --- | --- | --- | --- | --- | --- |
| **Measure 1** | |  | | **Measure 2** | | **BF₁₀** | | **W** | | **Rhat** | |
| Chinrest |  | - |  | No chinrest |  | 0.297 |  | 43.000 |  | 1.001 |  |
|  | | | | | | | | | | | |
| *Note.* Result based on data augmentation algorithm with 5 chains of 1000 iterations. | | | | | | | | | | | |

###
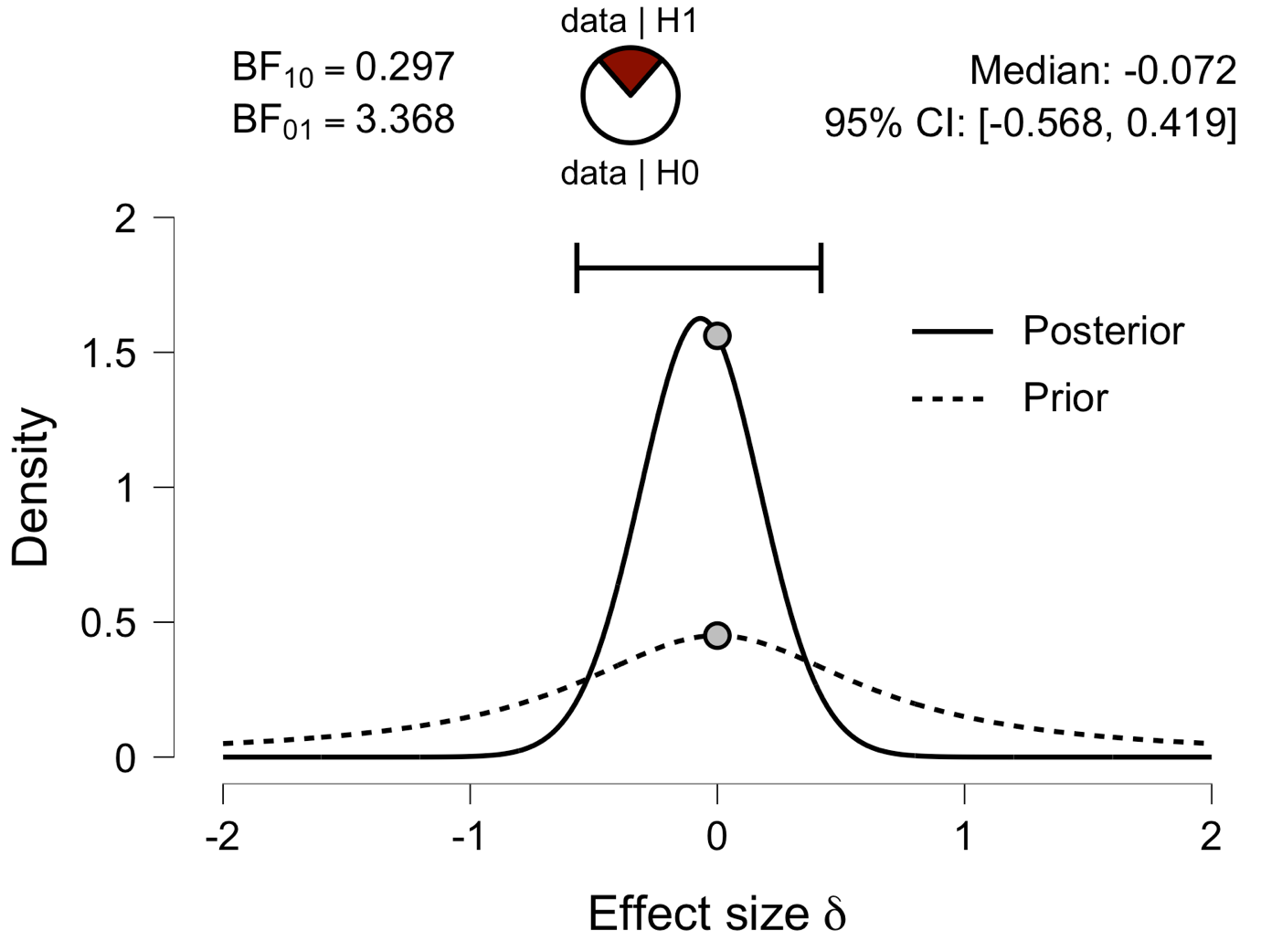
*Figure S3.* Inferential plots

**DV = Accuracy**

**Table S6.**

*Bayesian mixed model output for accuracy analyses reported in the paper (Experiment 1)*

| Model equation: **Vertical accuracy** ~ Dot position + Condition + (1 \| Participant) + (1 \| Trial) | | | | | | | | | | | | |
| --- | --- | --- | --- | --- | --- | --- | --- | --- | --- | --- | --- | --- |
|  | | | | | | 95% CI | | |  | | | |
|  | | Estimate | | SE | | Lower | | Upper | R-hat | | | ESS |
| Intercept |  | 1.41 |  | 0.12 |  | 1.18 |  | 1.64 |  | 1.001 |  | 707.88 |
| Condition (No chinrest – Chinrest) |  | 0.03 |  | 0.02 |  | - 0.02 |  | 0.07 |  | 1.000 |  | 8777.74 |
| Dot position (Peripheral – Central) |  | -0.29 |  | 0.05 |  | -0.40 |  | -0.19 |  | 1.001 |  | 1143.13 |
| Model equation: **Horizontal accuracy** ~ Dot position + Condition + (1 \| Participant) + (1 \| Trial) | | | | | | | | | | | | |
|  | | | | | | 95% CI | | |  | | | |
|  | | Estimate | | SE | | Lower | | Upper | R-hat | | | ESS |
| Intercept |  | 0.67 |  | 0.08 |  | 0.50 |  | 0.83 |  | 1.001 |  | 1933.51 |
| Condition (No chinrest – Chinrest) |  | -0.09 |  | 0.03 |  | -0.15 |  | -0.04 |  | 1.000 |  | 10100.57 |
| Dot position (Peripheral – Central) |  | 0.21 |  | 0.05 |  | 0.12 |  | 0.30 |  | 1.000 |  | 3457.35 |
| Model equation: **Global accuracy** ~ Dot position + Condition + (1 \| Participant) + (1 \| Trial) | | | | | | | | | | | | |
|  | | | | | | 95% CI | | |  | | | |
|  | | Estimate | | SE | | Lower | | Upper | R-hat | | | ESS |
| Intercept |  | 1.63 |  | 0.12 |  | 1.40 |  | 1.89 |  | 1.001 |  | 1555.84 |
| Condition (No chinrest – Chinrest) |  | -0.04 |  | 0.03 |  | -0.10 |  | 0.03 |  | 1.000 |  | 13034.49 |
| Dot position (Peripheral – Central) |  | -0.14 |  | 0.05 |  | -0.23 |  | -0.04 |  | 1.001 |  | 4511.00 |

*Note*. CI = Credible interval. All statistical analyses were computed in JASP (JASP Team, 2020), and default JASP priors were used. All tests are two-tailed.

**DV = Precision**

**Table S7.**

*Bayesian mixed model output for precision analyses reported in the paper (Experiment 1)*

| Model equation: **Vertical precision** ~ Dot position + Condition + (1 \| Participant) + (1 \| Trial) | | | | | | | | | | | | |
| --- | --- | --- | --- | --- | --- | --- | --- | --- | --- | --- | --- | --- |
|  | | | | | | 95% CI | | |  | | | |
|  | | Estimate | | SE | | Lower | | Upper | R-hat | | | ESS |
| Intercept |  | 0.28 |  | 0.02 |  | 0.244 |  | 0.31 |  | 1.001 |  | 1512.89 |
| Condition (No chinrest – Chinrest) |  | -0.01 |  | < 0.01 |  | -0.02 |  | -0.01 |  | 1.000 |  | 17279.64 |
| Dot position (Peripheral – Central) |  | -0.01 |  | 0.01 |  | -0.01 |  | 0.01 |  | 1.000 |  | 6998.78 |
| Model equation: **Horizontal precision** ~ Dot position + Condition + (1 \| Participant) + (1 \| Trial) | | | | | | | | | | | | |
|  | | | | | | 95% CI | | |  | | | |
|  | | Estimate | | SE | | Lower | | Upper | R-hat | | | ESS |
| Intercept |  | 0.25 |  | 0.01 |  | 0.22 |  | 0.28 |  | 1.000 |  | 1198.73 |
| Condition (No chinrest – Chinrest) |  | < 0.01 |  | < 0.01 |  | -0.01 |  | 0.01 |  | 1.000 |  | 11445.26 |
| Dot position (Peripheral – Central) |  | 0.01 |  | 0.01 |  | -4.57 e-4 |  | 0.02 |  | 1.000 |  | 4261.88 |
| Model equation: **Global precision** ~ Dot position + Condition + (1 \| Participant) + (1 \| Trial) | | | | | | | | | | | | |
|  | | | | | | 95% CI | | |  | | | |
|  | | Estimate | | SE | | Lower | | Upper | R-hat | | | ESS |
| Intercept |  | 0.36 |  | 0.02 |  | 0.32 |  | 0.40 |  | 1.001 |  | 1051.50 |
| Condition (No chinrest – Chinrest) |  | -0.01 |  | 0.01 |  | -0.02 |  | < 0.01 |  | 1.000 |  | 12772.36 |
| Dot position (Peripheral – Central) |  | < 0.01 |  | 0.01 |  | -0.01 |  | 0.02 |  | 1.000 |  | 5365.22 |

*Note*. CI = Credible interval. All statistical analyses were computed in JASP (JASP Team, 2020), and default JASP priors were used. All tests are two-tailed.

**Additional details**


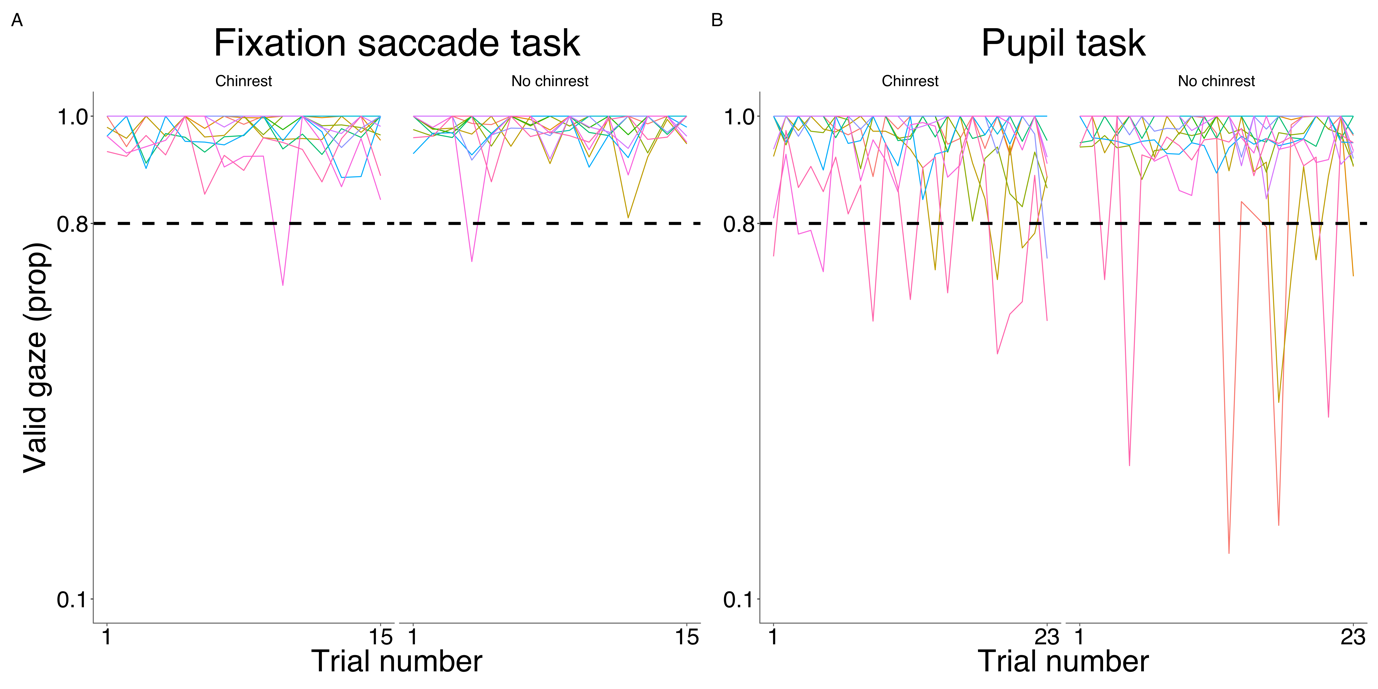


*Figure S4.* The proportion of valid/lost gaze across conditions (chinrest, no-chinrest) and tasks (Fixation-Saccade, PLR task). Coloured lines represent data loss over trials for each participant.


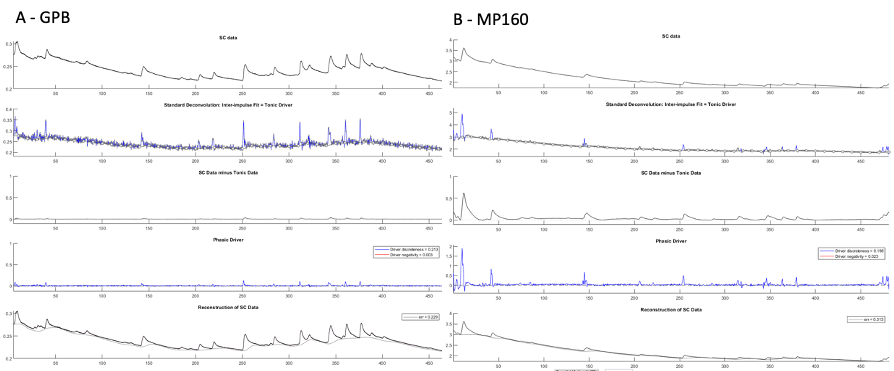


*Figure S5*. Sample plots of data from one representative participant in an equivalent portion of the task (the first block) from the Gazepoint Biometrics System (GPB) and the BIOPAC MP160.


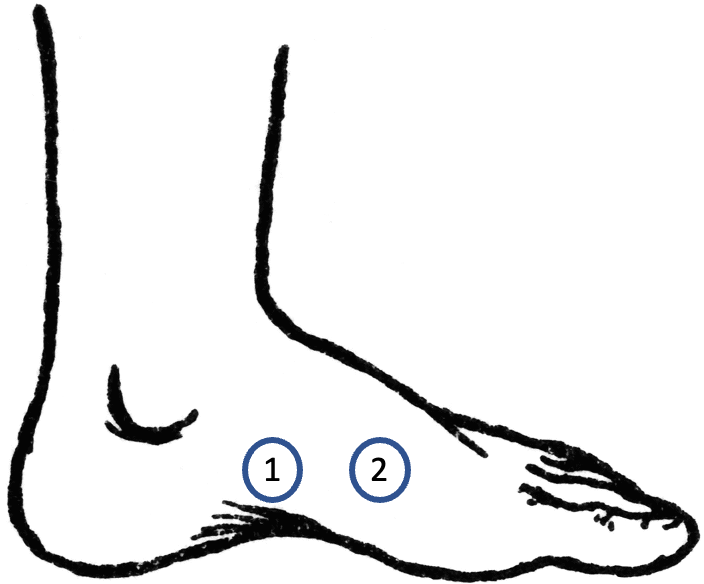


*Figure S6.* Diagram demonstrating where the BIOPAC foot sensors were attached.


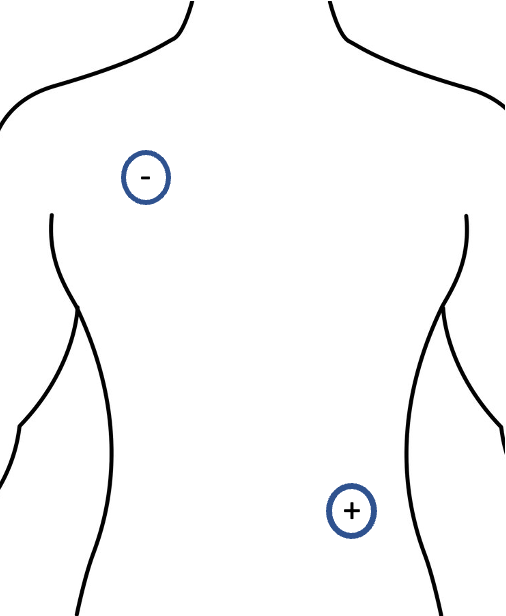


*Figure S7.* Diagram demonstrating where the BIOPAC torso sensors were attached (Points RA and LL).

**References**

JASP Team (2020). JASP (Version 0.14.1) [Computer software].
